# A loss-of-function *IFNAR1* allele in Polynesia underlies severe viral diseases in homozygotes

**DOI:** 10.1084/jem.20220028

**Published:** 2022-04-20

**Authors:** Paul Bastard, Kuang-Chih Hsiao, Qian Zhang, Jeremy Choin, Emma Best, Jie Chen, Adrian Gervais, Lucy Bizien, Marie Materna, Christine Harmant, Maguelonne Roux, Nicola L. Hawley, Daniel E. Weeks, Stephen T. McGarvey, Karla Sandoval, Carmina Barberena-Jonas, Consuelo D. Quinto-Cortés, Erika Hagelberg, Alexander J. Mentzer, Kathryn Robson, Boubacar Coulibaly, Yoann Seeleuthner, Benedetta Bigio, Zhi Li, Gilles Uzé, Sandra Pellegrini, Lazaro Lorenzo, Zineb Sbihi, Sylvain Latour, Marianne Besnard, Tiphaine Adam de Beaumais, Evelyne Jacqz Aigrain, Vivien Béziat, Ranjan Deka, Litara Esera Tulifau, Satupa‘itea Viali, Muagututi‘a Sefuiva Reupena, Take Naseri, Peter McNaughton, Vanessa Sarkozy, Jane Peake, Annaliesse Blincoe, Sarah Primhak, Simon Stables, Kate Gibson, See-Tarn Woon, Kylie Marie Drake, Adrian V.S. Hill, Cheng-Yee Chan, Richard King, Rohan Ameratunga, Iotefa Teiti, Maite Aubry, Van-Mai Cao-Lormeau, Stuart G. Tangye, Shen-Ying Zhang, Emmanuelle Jouanguy, Paul Gray, Laurent Abel, Andrés Moreno-Estrada, Ryan L. Minster, Lluis Quintana-Murci, Andrew C. Wood, Jean-Laurent Casanova

**Affiliations:** 1 Laboratory of Human Genetics of Infectious Diseases, Necker Branch, INSERM U1163, Necker Hospital for Sick Children, Paris, France; 2 St. Giles Laboratory of Human Genetics of Infectious Diseases, Rockefeller Branch, The Rockefeller University, New York, NY; 3 Paris Cité University, Imagine Institute, Paris, France; 4 Department of Pediatrics, Necker Hospital for Sick Children, Assistance Publique – Hôpitaux de Paris, Paris, France; 5 Starship Child Health, Auckland, New Zealand; 6 Department of Paediatrics: Child and Youth Health, Faculty of Medical and Health Sciences, University of Auckland, Auckland, New Zealand; 7 Murdoch Children’s Research Institute, Melbourne, Australia; 8 Clinical Immunogenomics Research Consortium Australasia, Sydney, Australia; 9 Institut Pasteur, Université de Paris, CNRS UMR2000, Human Evolutionary Genetics Unit, Paris, France; 10 Chair of Human Genomics and Evolution, Collège de France, Paris, France; 11 Paris Cité University, Paris, France; 12 Department of Infectious Diseases, Shanghai Sixth Hospital, Shanghai Jiaotong University, Shanghai, China; 13 Institut Pasteur, Université de Paris, Bioinformatics and Biostatistics Hub, Paris, France; 14 Department of Chronic Disease Epidemiology, Yale University School of Public Health, New Haven, CT; 15 International Health Institute, Department of Epidemiology, School of Public Health, Brown University, Providence, RI; 16 Department of Human Genetics, School of Public Health, University of Pittsburgh, Pittsburgh, PA; 17 Department of Biostatistics, School of Public Health, University of Pittsburgh, Pittsburgh, PA; 18 Department of Anthropology, Brown University, Providence, RI; 19 National Laboratory of Genomics for Biodiversity (LANGEBIO) - UGA, CINVESTAV, Irapuato, Guanajuato, Mexico; 20 Department of Biology, University of Oslo, Oslo, Norway; 21 Wellcome Centre for Human Genetics, University of Oxford, Oxford, UK; 22 Big Data Institute, Li Ka Shing Centre for Health Information and Discovery, University of Oxford, Oxford, UK; 23 MRC Weatherall Institute of Molecular Medicine, University of Oxford, Oxford, UK; 24 Unit of Cytokine Signaling, Pasteur Institute, INSERM U1224, Paris, France; 25 Institute for Regenerative Medicine and Biotherapy, Université Montpellier, INSERM, CNRS, Montpellier, France; 26 Laboratory of Lymphocyte Activation and Susceptibility to EBV Infection, INSERM UMR 1163, Imagine Institute, Paris, France; 27 Department of Neonatology, Centre Hospitalier de Polynésie Française, Papeete, French Polynesia; 28 Precision Cancer Medicine Team, Institut Gustave Roussy, Villejuif, France; 29 Pharmacology - Pharmacogenetic Department, Hopital Saint-Louis, Assistance Publique – Hôpitaux de Paris, Paris, France; 30 Department of Environmental and Public Health Sciences, College of Medicine, University of Cincinnati, Cincinnati, OH; 31 Tupua Tamasese Meaole Hospital, Apia, Samoa; 32 School of Medicine, National University of Samoa, Apia, Samoa; 33 Lutia i Puava ae Mapu i Fagalele, Apia, Samoa; 34 Ministry of Health, Apia, Samoa; 35 Queensland Children’s Hospital and University of Queensland, Brisbane, Queensland, Australia; 36 Tumbatin Developmental Services, Sydney Children’s Hospital, Randwick, New South Wales, Australia; 37 School of Women’s and Children’s Health, University of New South Wales, Sydney, New South Wales, Australia; 38 Department of Forensic Pathology, Auckland City Hospital, Auckland, New Zealand; 39 Clinical Geneticist, South Island Hub, Genetic Health Service, Christchurch, New Zealand; 40 Department of Virology and Immunology, LabPLUS, Auckland City Hospital, Auckland, New Zealand; 41 Department of Molecular Medicine and Pathology, Faculty of Medical and Health Science, University of Auckland, Auckland, New Zealand; 42 Molecular Pathology, Canterbury Health Laboratories, Christchurch, New Zealand; 43 The Jenner Institute, Nuffield Department of Medicine, University of Oxford, Oxford, UK; 44 Chemical Pathology and Genetics, Canterbury Health Laboratories, Christchurch, New Zealand; 45 Department of Clinical Immunology, Auckland City Hospital, Auckland, New Zealand; 46 Laboratory of Research on Infectious Vector-borne Diseases, Institut Louis Malardé, Papeete, French Polynesia; 47 Garvan Institute of Medical Research, Sydney, Australia; 48 St Vincent’s Clinical School, Faculty of Medicine and Health, UNSW Sydney, Sydney, New South Wales, Australia; 49 Department of Immunology and Infectious Diseases, Sydney Children’s Hospital, Randwick, New South Wales, Australia; 50 Howard Hughes Medical Institute, New York, NY

## Abstract

Globally, autosomal recessive IFNAR1 deficiency is a rare inborn error of immunity underlying susceptibility to live attenuated vaccine and wild-type viruses. We report seven children from five unrelated kindreds of western Polynesian ancestry who suffered from severe viral diseases. All the patients are homozygous for the same nonsense *IFNAR1* variant (p.Glu386*). This allele encodes a truncated protein that is absent from the cell surface and is loss-of-function. The fibroblasts of the patients do not respond to type I IFNs (IFN-α2, IFN-ω, or IFN-β). Remarkably, this *IFNAR1* variant has a minor allele frequency >1% in Samoa and is also observed in the Cook, Society, Marquesas, and Austral islands, as well as Fiji, whereas it is extremely rare or absent in the other populations tested, including those of the Pacific region. Inherited IFNAR1 deficiency should be considered in individuals of Polynesian ancestry with severe viral illnesses.

## Teaser

Bastard et al. report a loss-of-function *IFNAR1* variant that is surprisingly common (allele frequency >1%) in individuals of western Polynesian ancestry, while it is absent or extremely rare elsewhere. Homozygotes for this allele are prone to severe viral diseases.

## Introduction

Inborn errors of genes controlling the cellular response to type I IFNs underlie life-threatening viral diseases. *IFNAR1* and *IFNAR2* encode the two chains of the type I IFN receptor (Duncan et al., 2015; Hernandez et al., 2019); *JAK1* and *TYK2* encode the constitutively associated tyrosine kinases (Eletto et al., 2016; Minegishi et al., 2006); and *STAT1*, *STAT2*, and *IRF9* encode the three components of ISGF-3, a multimeric transcription factor that binds to IFN-stimulated response elements (ISREs) to mediate the induction of IFN-stimulated genes (ISGs; Dupuis et al., 2003; Hambleton et al., 2013; Hernandez et al., 2018). Autosomal recessive (AR) complete STAT1, STAT2, and IRF9 deficiencies impair cellular responses not only to type I IFNs, but also those to type II IFNs (for STAT1) and/or type III IFNs (for STAT1, STAT2, and IRF9). AR complete TYK2 deficiency impairs cellular responses to IL-12 and IL-23 more profoundly than the response to type I IFNs (Kreins et al., 2015), whereas no complete defect of JAK1 has been reported (Eletto et al., 2016). Patients with any of these four recessive inborn errors of immunity underlying a complete deficiency of cellular responses to type I IFNs are prone to various viral diseases, with AR STAT1 deficiency underlying the most diverse and severe set of viral diseases, including those caused by live attenuated virus (LAV) vaccines (Gothe et al., 2021; Poyhonen et al., 2019).

The crucial role of type I IFNs in susceptibility to LAV, herpes simplex virus 1 (HSV-1), and SARS-CoV-2 was revealed by the discovery of patients with AR IFNAR1 or IFNAR2 deficiency (Casanova and Abel, 2021). AR complete IFNAR1 deficiency was discovered in a 9-yr-old Iranian boy who suffered severe measles, mumps, and rubella (MMR) vaccine disease at the age of 1 yr (Hernandez et al., 2019). Nine other patients with suspected (*n* = 1) or confirmed (*n* = 8) IFNAR1 deficiency have been reported: a 14-yr-old child from Brazil with yellow fever virus (YFV) live vaccine–associated disease (Hernandez et al., 2019); a 2-yr-old child from Palestine with HSV-1 encephalitis (HSE); two cousins, aged 1 and 17 yr, with fatal complications following MMR vaccination and deafness following mumps, respectively (Bastard et al., 2021b); a 15-mo-old boy with severe disease following MMR vaccination (Gothe et al., 2022); a 13-yr-old boy from Saudi Arabia with critical COVID-19 pneumonia (Khanmohammadi et al., 2022); two adults, aged 26 and 38 yr, from Saudi Arabia and Turkey, with critical COVID-19 pneumonia (Zhang et al., 2020); and one 3-yr-old child from Iran with critical COVID-19 pneumonia and features of multisystem inflammatory syndrome (Abolhassani et al., 2022).

One patient with IFNAR2 deficiency and MMR disease has been described (Duncan et al. 2015), with two other patients more recently reported to have suffered from YFV vaccine or MMR disease (Bastard et al., 2021c; Passarelli et al., 2020). The clinical penetrance of IFNAR1 and IFNAR2 deficiencies for severe and life-threatening disease appears to be high for adverse reactions to LAV, including the YFV and MMR vaccines, and critical COVID-19 pneumonia (Bastard et al., 2021c; Duncan et al., 2015; Gothe et al., 2022; Hernandez et al., 2019; Khanmohammadi et al., 2022; Passarelli et al., 2020; Zhang et al., 2018). By contrast, penetrance seems to be lower for severe disease caused by at least some WT viruses, such as HSE (Bastard et al., 2021b). This is consistent with the surprisingly narrow range of severe and life-threatening viral illnesses in these patients, individually and collectively (Meyts and Casanova, 2021). However, it is not possible to draw any firm conclusion about the penetrance of IFNAR1 deficiency for most viral diseases at the moment, owing to the small number of patients diagnosed and the associated ascertainment bias.

The essential and nonredundant roles of human type I IFNs in antiviral defense were further delineated by the discovery of autoantibodies neutralizing type I IFNs, especially the 13 IFN-α and/or -ω, and less frequently IFN-β, in ≥15% of adults with life-threatening COVID-19 pneumonia (Bastard et al., 2021a; Bastard et al., 2020). These autoantibodies were also found to underlie adverse reactions to YFV vaccine in three adults (Bastard et al., 2021c). Interestingly, the patients with critical COVID-19 pneumonia or YFV vaccine disease, including many elderly patients, were previously healthy and had not suffered from unusually severe viral diseases. These findings are reminiscent of those for patients with inherited IFNAR1 or IFNAR2 deficiency. Together with our previous observation of previously healthy 26- and 38-yr-old IFNAR1-deficient patients with critical COVID-19, these findings raise the possibility that inborn errors of type I IFN responses might be clinically silent for many years, or even decades in some patients, until exposed to specific viral pathogens. We studied seven children from five unrelated kindreds of western Polynesian ancestry who developed severe or fatal complications following exposure to LAV and/or WT viruses.

## Results

### Life-threatening viral diseases in seven patients of western Polynesian ancestry

**Figure 1.**
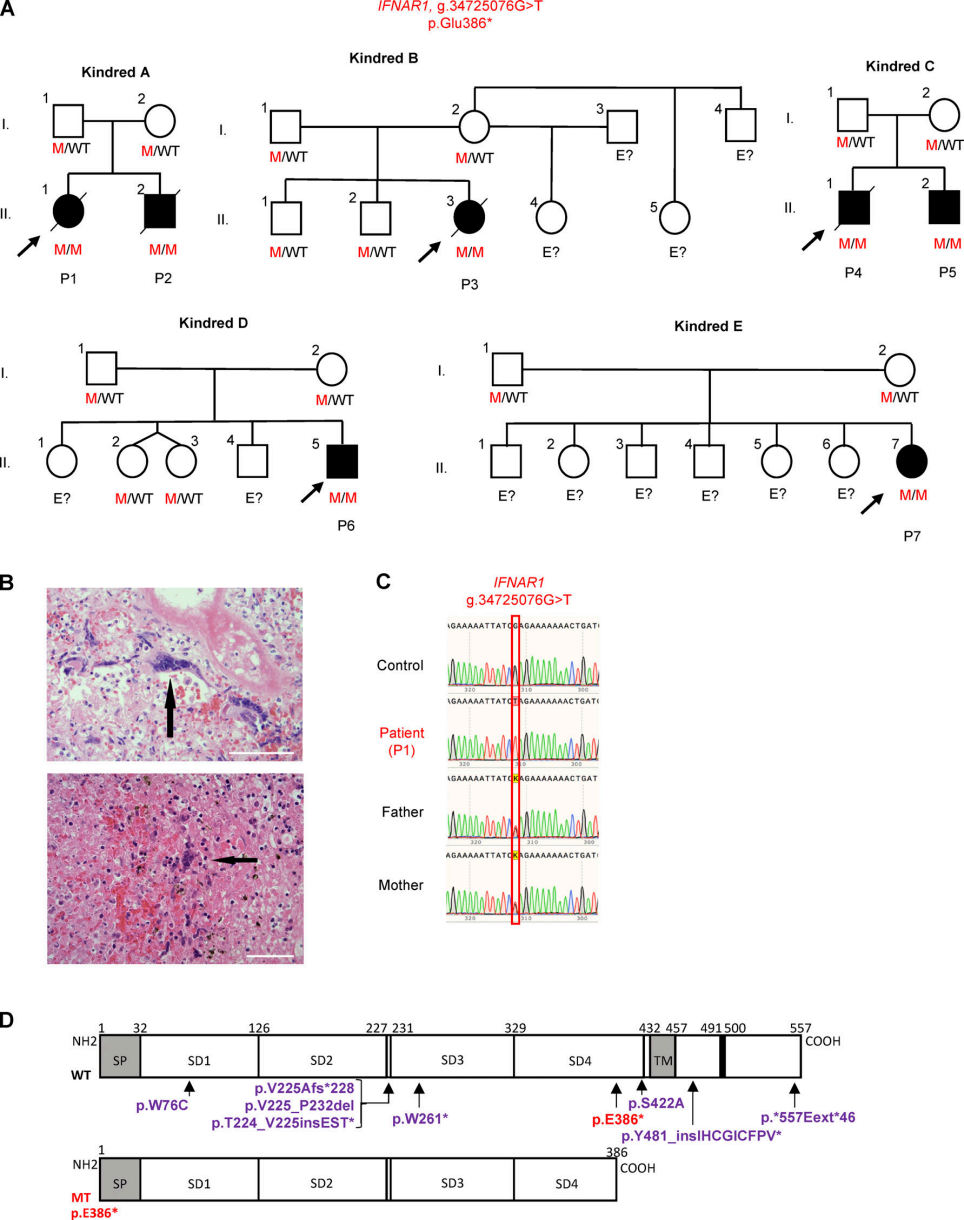
**Homozygous pLOF *IFNAR1* variant in Polynesians with adverse events after LAV vaccination. (A)** Family pedigree showing the segregation of the *IFNAR1* p.Glu386* allele in the seven patients from five kindreds. The proband is indicated by an arrow. E?, unknown *IFNAR1* genotype. **(B)** The giant multinucleated WFCs (identified by black arrows) in the lung (upper panel) and LNs (lower panel) of P1 are pathognomic of measles infection (Laksono et al., 2016). WFC was determined for the tonsils and adenoids during prodromal measles (Nozawa et al., 1994) and in the regional LNs after immunization and after fatal measles infection (Becroft and Osborne, 1980). Scale bar represents 400 µm. **(C)** Sanger sequencing results for *IFNAR1* in leukocyte gDNA from the patients, their parents, and healthy controls. **(D)** Schematic diagram of the WT and mutant (MT) IFNAR1 proteins. SD1–4, extracellular subdomains 1–4; SP, signal peptide; TM, transmembrane region. The mutation reported here is indicated in red, and the previously reported mutations are indicated in violet.

We studied seven children from five unrelated kindreds with severe or life-threatening clinical disease temporally associated with exposure to LAV and/or WT viruses (Fig. 1 A; Table 1, Tables S1, S2, S3, and S4). In brief, patient 1 (P1), from kindred A, was a previously healthy 12-mo-old girl of western Polynesian ancestry with non-consanguineous parents who presented with a pronounced injection site reaction 5 d after her first MMR vaccination. She had previously tolerated live attenuated bacillus Calmette-Guerin (BCG) vaccine and oral polio vaccine. Her condition deteriorated, with fever, thrombocytopenia, anemia, splenomegaly, marked hyperferritinemia, dyslipidemia, coagulopathy, and multiorgan failure, but with normal lymphocyte subsets. She was diagnosed with and treated for hemophagocytic lymphohistiocytosis (HLH) on day 14, but she died 18 d after MMR vaccination, at which time positive PCR results were obtained for vaccine-strain MMR viruses and human herpesvirus 6 (HHV6) on whole blood and a nasopharyngeal swab. Postmortem examination confirmed extensive hemophagocytosis and abnormal histiocytic infiltrates and revealed widespread giant multinucleate Warthin–Finkeldey cells (WFCs) associated with measles infection (Fig. 1 B; Nozawa et al., 1994). The patient’s older brother (P2) had also died at 12 mo of age, 21 d after his first MMR vaccination, with a similar clinical presentation.

**Table 1.** Clinical description of patients after exposure to MMR/V

Patient	P1	P2	P3	P4	P5	P6	P7
**Kindred**	A	A	B	C	C	D	E
Age at presentation (mo)	12	12	15	13	16	14	NA
Age at diagnosis of IFNAR1 deficiency	–^a^	–^a^	17 mo	13 mo	7 yr	4 yr	13 yr
**Level of care (post-exposure onset day)** ^b^							
Hospitalization, secondary	5	2	11	NA	NA	25	NA
Hospitalization, tertiary	14	NA	60	10	17	NA	NA
Hospitalization, PICU	14	NA	60	14	NA	NA	NA
**Clinical presentation (post-exposure onset day)** ^b^							
Localized injection site reaction	5	NR	NR	10	NR	NR	NA
Acute neurological clinical manifestations	10	Y	11	14	Y	25	NA
Acute hyperinflammation	14	Y	17	14	Y	NR	NA
Acute respiratory clinical manifestations	15	Y	62	14	N	NR	NA
Death	18	21	72	NA^c^	NA	NA	NA
Other	NA	NA	Knee + ankle joint effusions, day 24	NA	NA	NA	NA
**Other clinical features**							
Infections and comorbidities before dose 1 of MMR/V vaccination	NA	NA	EV meningitis; CNS structural anomalies	Premature birth (24 wk gestation), CLD, IVH, 2× bronchiolitis admissions <7 d	Tetralogy of Fallot (repaired at age 6 mo), 4× bronchiolitis admissions <7 d	NA	Hib sepsis/meningitis; RSV LRTI
Infections diagnosed after dose 1 of MMR/V vaccination	NA	NA	NA	Fatal RSV ARDS (+4 mo)	EV meningitis (+8 mo); RSV LRTI (+9 mo)	RSV, HPIV-3, RV, Bocavirus LRTI, ECMO (+9 mo)	LRTI, HFOV

ARDS, acute respiratory distress syndrome; CLD, chronic lung disease; day 1, day of exposure to LAV; HFOV, high-frequency oscillatory ventilation; IVH, intraventricular hemorrhage; LRTI, lower respiratory tract infection; N, no; NA, not applicable; NR, not reported; Y, yes.

a Diagnosed postmortem.

b MMR administered on day 1.

c Died from ARDS due to RSV 4 mo later.

Patient 3 (P3), from kindred B, was a 15-mo-old girl born to non-consanguineous parents who were also of western Polynesian ancestry, predominantly of Tongan and Niuēan descent; one great-great-grandfather was New Zealand Māori. P3 had presumed enteroviral meningoencephalitis at the age of 2 mo and a mild developmental delay, but otherwise normal neurological function. She presented with encephalopathy 11 d after initial exposure to the LAV in the MMR/V vaccine. She developed HLH-like symptoms of fever, hyperferritinemia, high levels of soluble CD25 (sCD25), organomegaly, and lymphadenopathy, and her bone marrow displayed signs of hemophagocytosis. She also developed bilateral arthritis. Steroid treatment was initiated, and the patient then developed PCR-positive cutaneous varicella reactivation that responded to acyclovir. Her peripheral blood was subsequently tested positive, by PCR, for the vaccine-strain measles virus. Retrospective PCR tests on joint fluid were positive for mumps viruses, and those on cerebrospinal fluid (CSF) were positive for vaccine-strain measles and mumps viruses 8 wk after MMR/V vaccination. Encephalopathy persisted, and P3 deteriorated further, dying 72 d after initial presentation.

Patient 4 (P4), from kindred C, was a 13-mo-old boy born at 24 wk of gestation to non-consanguineous parents of Niuēan ancestry. Premorbid conditions included stable chronic lung disease of prematurity and mild developmental delay without seizure disorder. 14 d after receiving the first dose of MMR vaccine, P4 developed status epilepticus, encephalopathy, and acute respiratory distress syndrome. This presentation was accompanied by fever, thrombocytopenia, anemia, hepatomegaly, hepatitis, hyperferritinemia, and high levels of sCD25. PCR on CSF was positive for vaccine-strain mumps virus, and PCR on peripheral blood was positive for vaccine-strain measles and mumps viruses on days 19–22 after MMR vaccination. P4 had a protracted recovery, remaining hospitalized for 4 mo. However, during home leave he acquired a respiratory tract infection, testing positive by PCR for respiratory syncytial virus (RSV), and he died, despite pediatric intensive care. P4 had a significant family history, as his older brother (P5), now aged 7 yr, was hospitalized for aseptic meningoencephalitis and features of hyperinflammation, initially diagnosed as atypical Kawasaki disease, 17 d after receiving the first dose of MMR vaccine, at the age of 16 mo. He was treated with intravenous cefotaxime, acyclovir, intravenous immunoglobulin, and oral aspirin. He then developed and recovered from meningitis due to an enterovirus (PCR-positive) at 2 yr of age, and he suffered from recurrent viral infections of the respiratory tract in early childhood. He tolerated his second dose of MMR vaccine without complications. P5 has no significant neurocognitive deficit at the age of 7 yr, but he suffers from unilateral sensorineural hearing loss, reminiscent of the sequelae of mumps virus infection.

Patient 6 (P6), from kindred D, is a 4-yr-old boy born to Niuēan and Tongan parents. He suffered recurrent viral pneumonia in the first year of life, but was otherwise reported to be developing normally. At the age of 14 mo, he received the MMR vaccine, and, 5 d later, he developed fever, lymphadenopathy, a generalized rash, and symptoms of viral encephalitis associated with CSF pleocytosis. Brain magnetic resonance imaging (MRI) findings were normal and, 4 wk after MMR vaccination, a PCR on nasopharyngeal aspirate (NPA) was positive for measles virus and negative for all other viruses tested. Following recovery, there were concerns about new-onset gross motor, hearing, and language delay. At the age of 21 mo, P6 developed viral pneumonia associated with positive PCR results for rhinovirus, parainfluenza, RSV, and bocavirus on NPA. He required venovenous extracorporeal membranous oxygenation (ECMO). He has since been diagnosed with profound bilateral sensorineural hearing loss requiring cochlear implants, global developmental delay (315.8 *Diagnostic and Statistical Manual of Mental Disorders, Fifth Edition* [DSM-5]), and autistic spectrum disorder (299.00 DSM-5), and he currently requires substantial support.

Patient 7 (P7), from kindred E, is a 13-yr-old girl born at term, in New Zealand, to non-consanguineous parents of Samoan ancestry, following an uneventful pregnancy. At 10 mo of age she presented with hemophilus influenza type B (Hib) bacteremia and meningitis. This illness was complicated by seizures and bilateral infected subdural hygromas requiring multiple washouts. This illness was probably due, in part, to the patient not having been vaccinated before this infection. At the age of 12 mo, 2 wk after her discharge from hospital following her Hib illness, she again presented with rapidly progressive respiratory and multiple-organ failure requiring 9 d of ECMO support. RSV was identified on PCR and immunofluorescence analyses. P7 moved to Australia at the age of 3 yr. She received the MMR vaccine at 4 yr of age, and the MMR/V vaccine 6 mo later, without complications. She remained relatively well until she presented at the age of 7 yr with acute respiratory distress syndrome and multiorgan failure requiring inotropic support. She was intubated for a total of 33 d and required escalation to high-frequency oscillation ventilation and nitric oxide treatment. Imaging showed extensive air-space opacification and a background of extensive bronchiectasis. No causal organism was identified.

LAVs were associated with severe complications in six of the seven children, three of whom died. The remaining patient tolerated LAV exposure. Features of hyperinflammation were present in five patients, possibly due to excessive production of cytokines other than type I IFNs (Gothe et al., 2022; Passarelli et al., 2020).

### A homozygous predicted to be loss-of-function (pLOF) variant of *IFNAR1* in the seven patients

**Figure S1.**
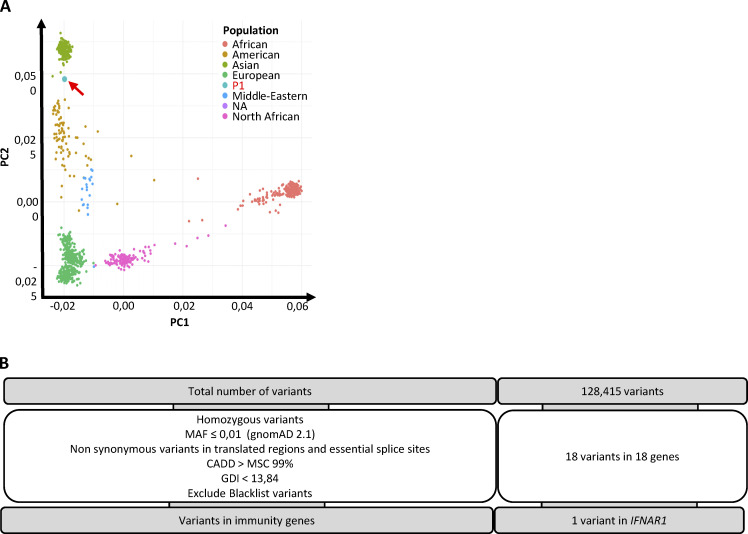
**Genetic analysis of P1. (A)** Principal component analysis (PCA) for P1. Although there are no Polynesians included in the PCA for triangulation of P1’s ancestry, individuals of Polynesian ancestry often locate on visualizations of the first and second principal components adjacent to East Asian samples (Minster et al., 2016). **(B)** Filtering criteria used for the single-nucleotide variant (SNV) analysis of WES results for P1. CADD, combined annotation-dependent depletion; GDI, gene damage index.

**Figure S2.**
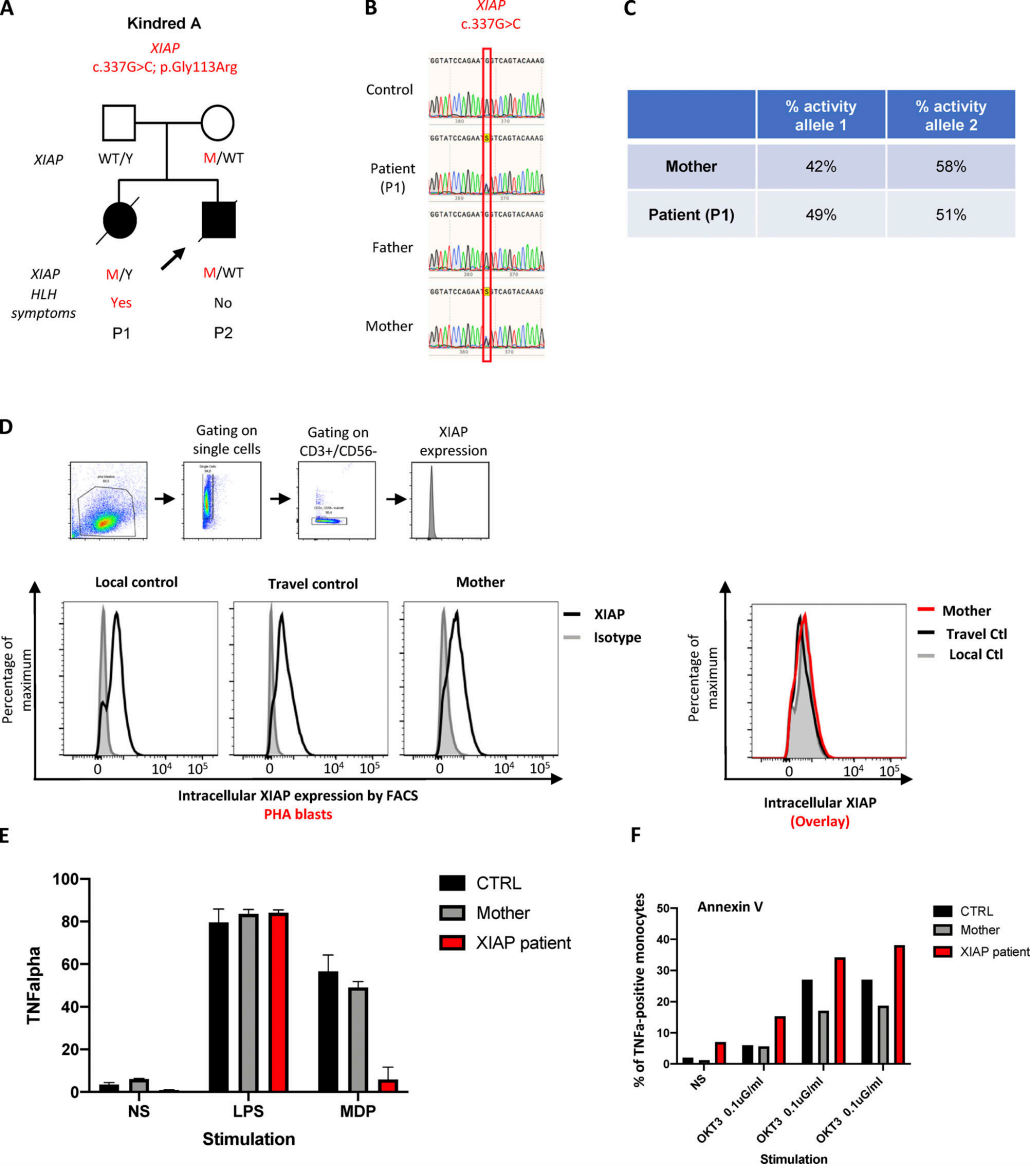
***XIAP***** p.Gly113Arg, carried by P1 and P2, is neutral. (A)** Pedigrees of families with *XIAP* variant (kindred A, P1, and P2 shown). Black boxes represent subjects affected by HLH-like. Diagonal bars indicate deceased subjects. **(B)** Sanger sequencing results for *XIAP* for P1 and P2 of kindred A, the parents, and healthy control leukocyte gDNA. **(C)** Lyonization in P1’s and mother’s gDNA. **(D)** Normal expression of XIAP by flow cytometry in CD3^+^ cells, a control, the mother of P1, and a XIAP-deficient patient. **(E)** Normal production of TNF-α in response to NOD2 stimulation by muramyl dipeptide (MDP) in monocytes from a control and the mother of P1, while it is defective in a XIAP-deficient patient. Bar graphs showing percentage of monocytes expressing TNFα from intracellular staining data. **(F)** Normal activation-induced cell death in response to anti-CD3, assessed by Annexin V staining, in T cells from a control and the mother of P1, while it is increased in a XIAP-deficient patient.

We performed whole-exome sequencing (WES) on P1. Principal component analysis suggested that the patient was of possible Polynesian ancestry (Fig. S1 A), and WES data confirmed that the parents were non-consanguineous. We analyzed the WES results, focusing on known monogenic defects compromising cellular responses to type I IFNs (*IFNAR1*, *IFNAR2*, *JAK1*, *TYK2*, *STAT1*, *STAT2*, and *IRF9*; Bastard et al., 2021b; Duncan et al., 2015; Duncan et al., 2021; Dupuis et al., 2003; Eletto et al., 2016; Hambleton et al., 2013; Hernandez et al., 2019; Hernandez et al., 2018; Minegishi et al., 2006). We searched for compound heterozygous or homozygous single-nucleotide variants or large deletions (copy number variants; Fig. S1 B). We also analyzed all genes underlying known inborn errors of immunity (Bousfiha et al., 2020; Notarangelo et al., 2020; Tangye et al., 2020) and searched for homozygous or compound heterozygous variants of any of the protein-coding genes considered that had been pLOF. We found that P1 carried a homozygous variant of *IFNAR1* (GRCh37 NC_000021.8:g.34725076G>T), leading to the creation of a premature stop codon, p.Glu386*. P1 also carried a heterozygous missense variant of *XIAP*, GRCh37 NC_000023.10:g.123019849G>C, p.(Gly113Arg), which was predicted to be benign and was confirmed experimentally to be biochemically neutral (Fig. S2, A–H). Sanger sequencing of *IFNAR1* in the index patient and her parents was consistent with an AR mode of inheritance (Fig. 1 C). We also sequenced *IFNAR1* in the other six affected patients (P2–P7) by Sanger sequencing and next-generation sequencing methods, as part of clinical care. We found that all six patients were homozygous for the same *IFNAR1* variant as P1. This pLOF variant is predicted to encode a truncated protein, lacking the transmembrane and intracellular domains (Fig. 1 D). These findings suggest that the seven patients had AR IFNAR1 deficiency, which was causal for the severe or fatal adverse events temporally associated with exposure to LAV and/or WT viruses.

### The p.Glu386* IFNAR1 protein is not expressed at cell surface and is loss-of-function

**Figure 2.**
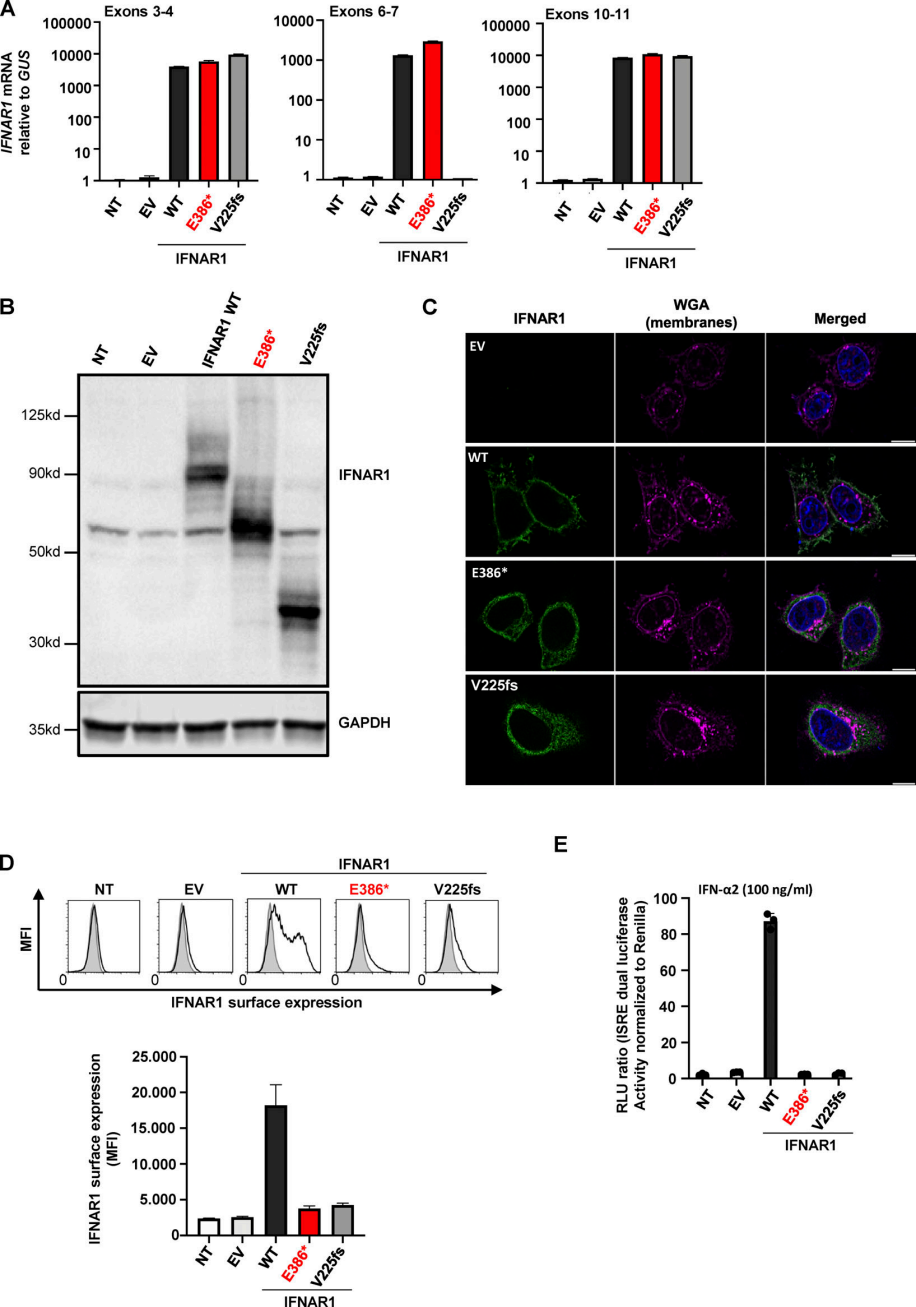
The *IFNAR1* p.Glu386* variant results in a truncated protein that is not expressed at cell surface and is loss-of-function. (A) *IFNAR1* mRNA levels, determined by RT-qPCR, in HEK293T cells transiently transfected with WT or MT *IFNAR1* cDNA constructs; β glucuronidase (GUS) was used as an expression control. EV, empty vector; NT, nontransfected; p.V225fs variant from a previously reported *IFNAR1*^−/−^ patient. Error bars indicate SD. **(B)** Western blot of IFNAR1 in HEK293T cells transiently transfected with WT and mutant *IFNAR1* cDNA constructs. An antibody recognizing the IFNAR1 protein was used. GAPDH was used as a loading control. One blot representative of two independent experiments is shown. **(C)** Immunofluorescence staining as assessed by confocal microscopy in HeLa cells transiently transfected with *IFNAR1* cDNA constructs. An antibody against the N-terminus of IFNAR1 was used (green), and membranes were stained with wheatgerm agglutinin (WGA; purple). The nuclei were stained with DAPI (blue). Scale bar represents 10 µm. The images shown are representative of two independent experiments. **(D)** Graphical representation of extracellular FACS staining and the mean fluorescence intensity (MFI) for IFNAR1 in HEK293T cells transiently transfected with *IFNAR1* cDNA constructs, performed with an antibody recognizing the N-terminus of the protein. Cells were not permeabilized. Results representative of three independent experiments are shown. Error bars indicate the SD. **(E)** Luciferase activity after IFN-α2 stimulation in *IFNAR1*^−/−^ HEK293T cells generated with CRISPR/Cas-9 technology and transiently transfected with WT or MT *IFNAR1* cDNA constructs. The bars represent the means and SEM of the results obtained in three independent experiments. Ctrl, control; RLU, relative light units. Source data are available for this figure: SourceData F2.

We then studied the expression of WT or p.Glu386* *IFNAR1* following the transient transfection of HEK293T cells with plasmids containing the corresponding cDNAs. As a control, we used the previously reported p.Val225Alafs*228 *IFNAR1* variant (referred to here as p.V225fs; Hernandez et al., 2019). Quantitative RT-PCR (RT-qPCR) revealed that *IFNAR1* mRNA levels were similar for the WT and p.Glu386* forms (Fig. 2 A). Western blotting of extracts of these cells with an antibody specific for the N-terminal region of IFNΑR1 revealed a truncated protein for p.Glu386* IFNΑR1, migrating at a lower molecular weight than the WT IFNΑR1 (Fig. 2 B). We then analyzed cell surface levels of WT and p.Glu386* IFNAR1 by FACS and confocal microscopy. In these overexpression systems, p.Glu386* IFNAR1 was not detected at the plasma membrane (Fig. 2, C and D). Finally, we used the WT or p.Glu386* *IFNAR1* cDNA to transfect *IFNAR1-*deficient HEK293T cells generated by CRISPR/Cas9-mediated gene editing. Following transfection with a reporter gene containing five ISREs and stimulation with IFN-α2, luciferase activity levels in the cells expressing WT IFNAR1 were 10 times higher than those in nontransfected cells or cells transfected with an empty vector. By contrast, HEK293T cells transduced with the p.Glu386* variant displayed a total absence of luciferase activity in response to IFN-α2, consistent with the response of cells transduced with the p.V225fs *IFNAR1* variant (Fig. 2 E). Thus, the p.Glu386* *IFNAR1* variant encodes a truncated protein that is LOF and not expressed on the cell surface. These in vitro studies of the p.Glu386* allele, in isolation and by overexpression, also suggest that all seven patients had AR IFNAR1 deficiency.

### The cells from one patient do not express IFNAR1 and do not respond to type I IFNs

**Figure 3.**
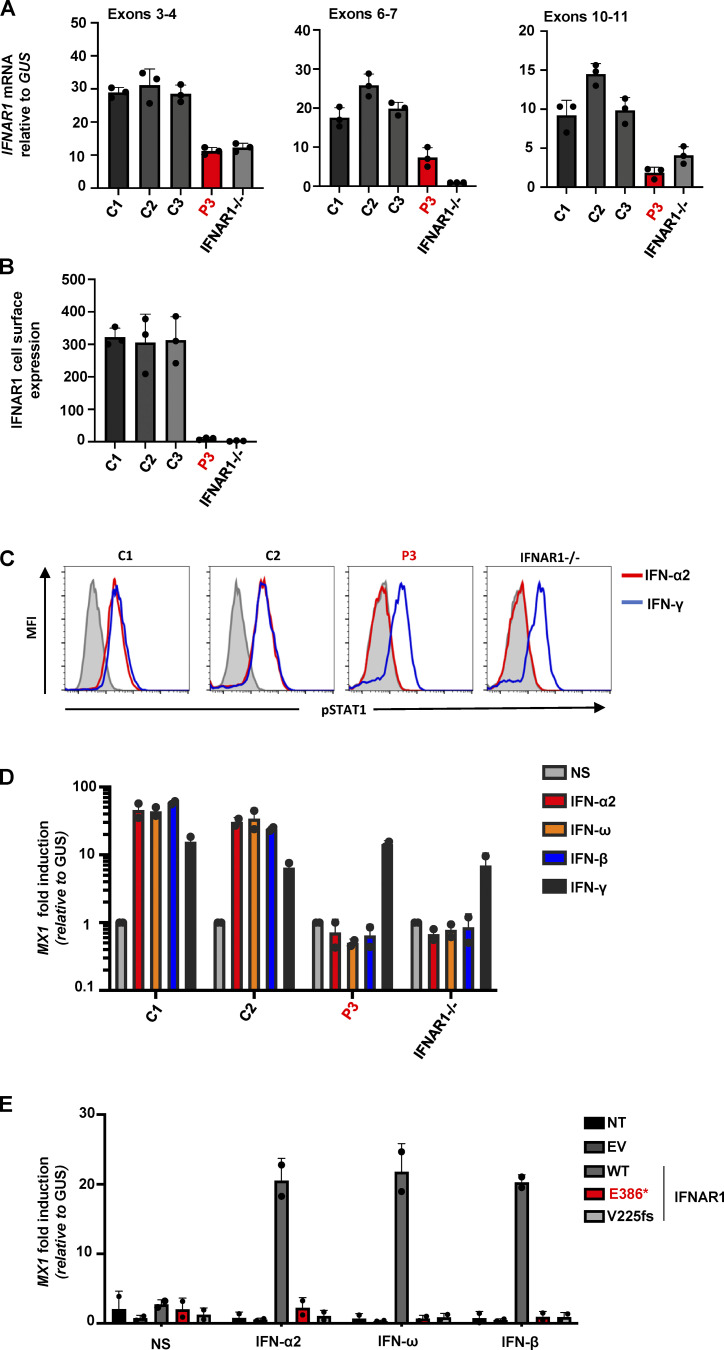
**Patient SV40-fibroblasts do not express IFNAR1 at the cell surface and do not respond to IFN-α/β. (A)** *IFNAR1* mRNA levels in SV40-fibroblasts from three healthy controls (C1, C2, C3), P3, and the previously reported p.V225fs IFNAR1^−/−^ patient; *GUS* was used as an expression control. Mean values and SD from two independent experiments, each with technical duplicates, are shown. **(B)** Mean fluorescence intensity (MFI) following extracellular FACS staining of IFNAR1 in SV40-fibroblasts from three healthy controls (C1, C2, and C3), P3, and a previously reported IFNAR1^−/−^ patient. Cells were not permeabilized. An antibody recognizing the N-terminal part of the protein was used. **(C)** Intracellular FACS staining of p-STAT1 in SV40-fibroblasts stimulated with 100 ng/ml IFN-α2, or IFN-γ for 15 min, in two healthy controls (C1 and C2), P3, and a previously reported IFNAR1^−/−^ patient. **(D)** Fold-change in *MX1* mRNA levels after 6 h of stimulation with IFN-α2b, IFN-β, IFN-ω, or IFN-γ, in SV40-fibroblasts from two healthy controls (C1 and C2), P3, and IFNAR1^−/−^ patients. The *GUS* housekeeping gene was used as an expression control. The mean and SD values from two independent experiments are shown. **(E)** Fold-change in *MX1* mRNA levels after stimulation for 6 h with IFN-α2b, IFN-β, or IFN-ω in SV40-fibroblasts from P3, transfected on the previous day with empty vector, MT, or WT IFNAR1 plasmids. The *GUS* housekeeping gene was used as an expression control. Mean and SD values from two independent experiments are shown.

**Figure S3.**
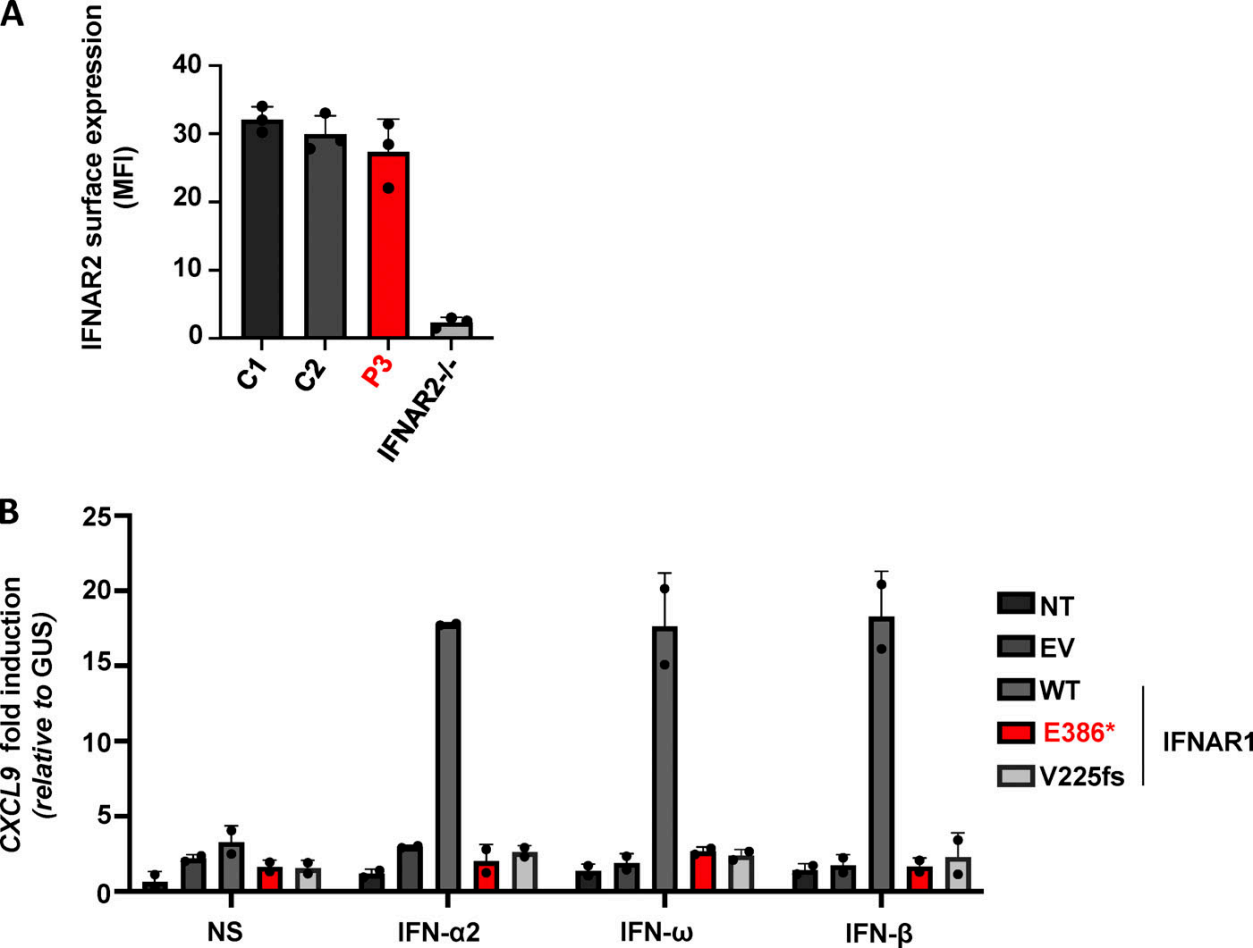
**Normal IFNAR2 expression and impaired response to type I IFNs in the patient's cells. (A)** Mean fluorescence intensity (MFI) following extracellular FACS staining of IFNAR2 in SV40-fibroblasts from two healthy controls (C1 and C2), P3, and a previously reported IFNAR2^−/−^ patient. Cells were not permeabilized. An antibody recognizing the N-terminal part of the protein was used. **(B)** Fold-change in *CXCL9* mRNA levels after stimulation for 6 h with IFN-α2b, IFN-β, or IFN-ω in SV40-fibroblasts from P3, transfected on the previous day with empty vector, MT, or WT IFNAR1 plasmids. The GUS housekeeping gene was used as an expression control. Mean and SD values from two independent experiments are shown.

We then generated and tested SV40-transformed dermal fibroblasts for P3. We first showed, by RT-PCR, that they produced only small amounts of *IFNAR1* mRNA (Fig. 3 A). No IFNΑR1 protein was detected, by FACS, at the cell surface (Fig. 3 B), whereas IFNΑR2 levels were normal (Fig. S3 A). The binding of type I IFNs to the receptor complex leads to the activation of the constitutively associated TYK2 and JAK1 tyrosine kinases, and then to the phosphorylation of STAT1 and STAT2, which associate with IRF9 to form the trimeric ISGF3 complex. The activated ISGF-3 complex then migrates to the nucleus, where it promotes the expression of ISGs. We therefore assessed the cellular responses of the patient’s cells to type I IFN (100 ng/ml of IFN-α2). STAT1 phosphorylation in response to IFN-α2 stimulation was completely abolished in the patient’s fibroblasts, as reported for a previously described IFNAR1-deficient patient (Fig. 3 C; Hernandez et al., 2019). We then confirmed the defective response to type I IFNs by measuring the induction of transcription for the *MX1* ISG. Stimulation with type I IFNs (IFN-α2, IFN-ω, and IFN-β) induced *MX1* and *CXCL9* mRNA in control SV40-fibroblasts, but this response was abolished in SV40-fibroblasts from P3 (Fig. 3 D and Fig. S3 B). Importantly, STAT1 phosphorylation and *MX1* induction in response to IFN-γ were intact in the SV40-fibroblasts of P3 (Fig. 3, C and D). Finally, the lack of IFN-α/ω/β–mediated ISG induction in SV40-fibroblasts from P3 was rescued by the overexpression of WT, but not p.Glu386* or p.V225fs IFNAR1 (Fig. 3 E). Overall, the patient’s cells had an abolished response to type I IFNs but responded normally to type II IFN. As all seven patients carried the same *IFNAR1* variant in the homozygous state, these findings are consistent with the conclusion that all these patients had AR complete IFNAR1 deficiency.

### The p.Glu386* *IFNAR1* variant is found at an allele frequency of 1.25% in Samoa

**Figure S4.**
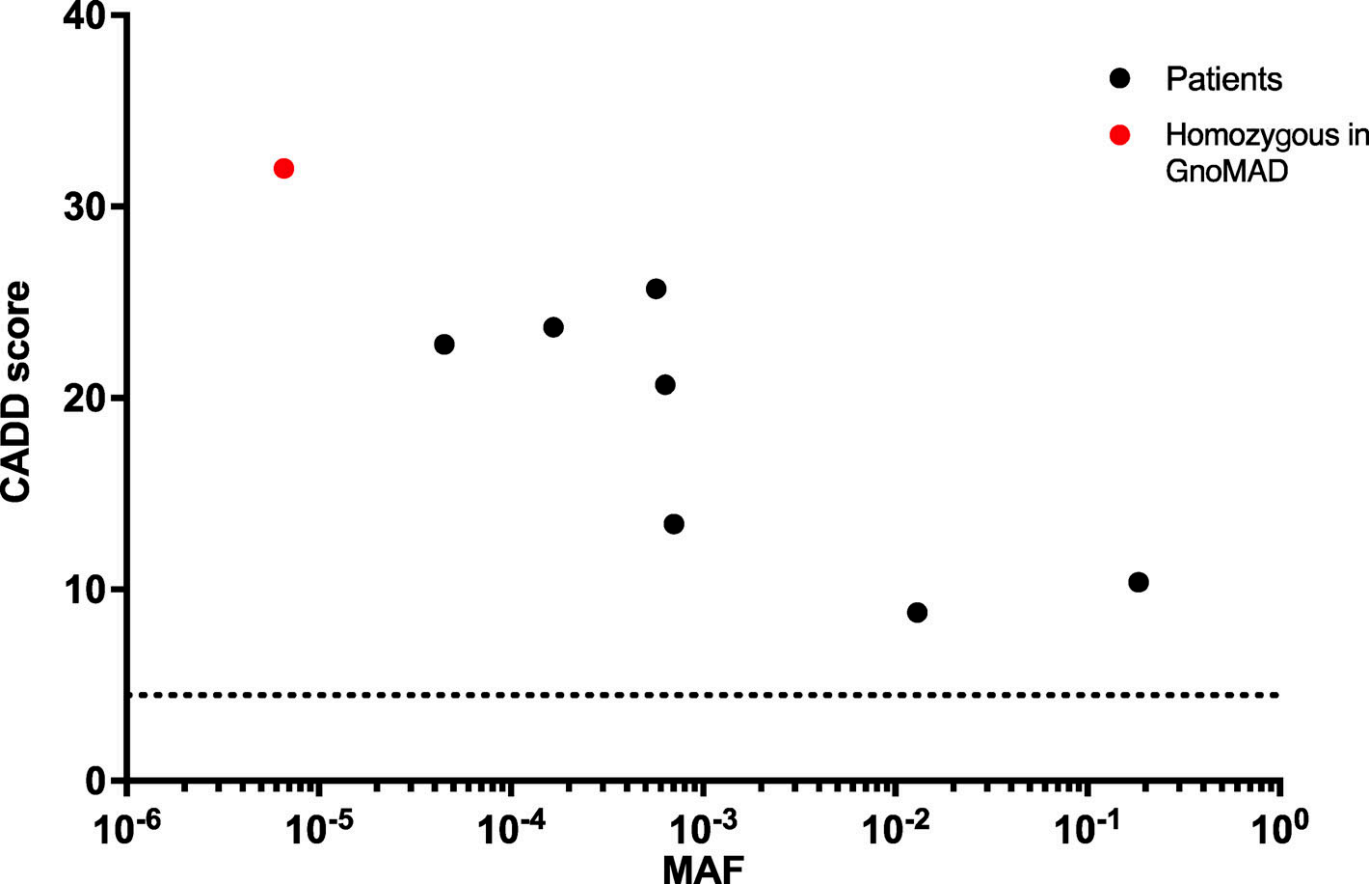
**Population genetics of homozygous coding missense IFNAR1 variants present in gnomAD v2.1.** The patient’s variant is shown in red, whereas the variants present in gnomAD are shown in black. Dotted line represents the gene damage index. CADD, combined annotation-dependent depletion.

**Figure 4.**
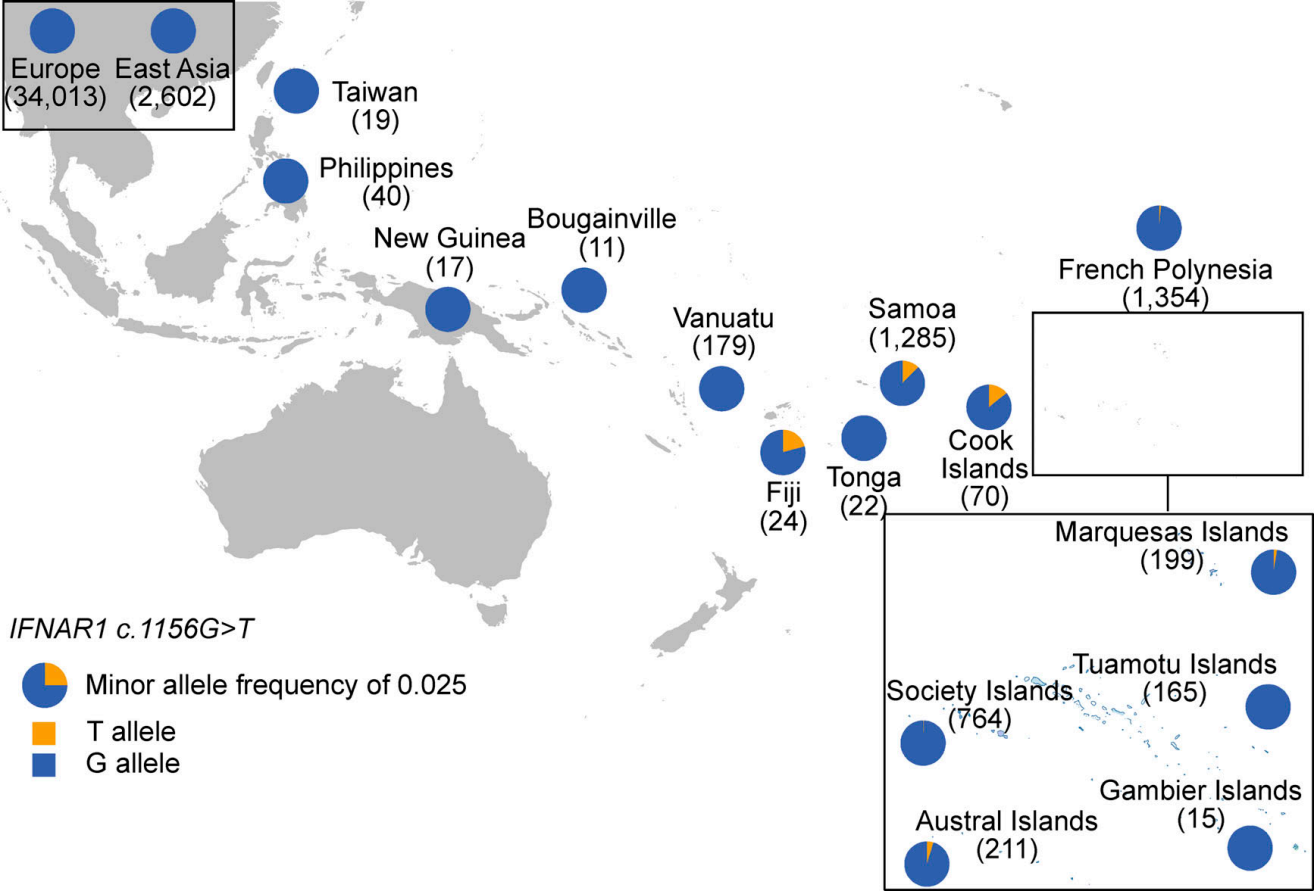
The p.Glu386* variant is extremely rare or absent in most regions of the world tested, but frequent in western Polynesia. Map showing the frequency distribution of the two alleles (c.1156G>T) in different Pacific and continental populations (Europeans [non-Finnish] and East Asians from gnomADv3.2.1; Karczewski et al., 2020), and Pacific populations including Taiwanese indigenous peoples, Philippine populations, and ni-Vanuatu (Choin et al., 2021); New Guineans and Bougainville islanders (Bergstrom et al., 2020); Polynesians from Samoa (the Soifua Manuia study; Harris et al., 2020); and populations from Fiji, Tonga, and the Cook, Society, Austral, Tuamotu, Gambier, and Marquesas Islands. To aid visualization of the variant distribution, a factor 10 correction has been applied to allele frequencies, so that a full pie chart corresponds to MAF = 10% and a quarter of a pie chart to MAF = 2.5% (as denoted in the schematic legend).

We then investigated the population genetics landscape of the variant. The *IFNAR1* g.34725076G>T variant is absent from gnomAD v2, but is present in one heterozygous individual in v3.1 of this public database (https://gnomad.broadinstitute.org/variant/21-33352770-G-T?dataset=gnomad_r3). No pLOF variants of *IFNAR1* in the homozygous state are present in this database, which includes only seven missense variants in homozygosity (Fig. S4). The five kindreds we report were unrelated to each other, and the parents were all non-consanguineous and of western Polynesian origin. Western Polynesia is a geographic area thought to be the homeland of the ancestral Polynesian society (Addison and Matisoo-Smith, 2010; Vinton Kirch, 2017). It includes Tonga, the Independent State of Samoa, American Samoa, and Niuē (Fig. 4). We assessed the prevalence of the *IFNAR1*:p.Glu386* LOF variant in various populations across the Pacific. We analyzed a cohort of 1,285 adult Samoans from the Soifua Manuia (“good health” in Samoan) study who had undergone whole-genome sequencing as part of the Trans-Omics in Precision Medicine (TOPMed) Whole-Genome Sequencing Program (Harris et al., 2020; Taliun et al., 2021). In this cohort, *IFNAR1*:c.1156G>T p.(Glu386*) (rs201609461) had a frequency of 0.0125 (95% CI: 0.0085, 0.0175) Note that the rs201609461 designation applies to the two observed alternative alleles: G>T and G>A. Only the frequency of G>T is of relevance here. We observed 1,253 G-allele homozygotes, 32 heterozygotes, and no T-allele homozygotes. We found no evidence for a deviation of the distribution of genotypes from Hardy–Weinberg equilibrium (exact test, P = 1). Under Hardy–Weinberg assumptions, the carrier frequency, as estimated from the sequenced alleles, is 0.0246, or 1:41 individuals, and the estimated frequency of the homozygous genotype is 0.000155, or 1:6,450 individuals. A bias in the allele frequencies calculated is possible, due to the presence of related individuals. We therefore also examined a subset of 827 unrelated individuals among the Soifua Manuia study. In this subset, G>T had a frequency of 0.0109 (95% CI: 0.0065, 0.0171), and there were 809 G-allele homozygotes, 18 heterozygotes, and no T-allele homozygotes.

### The distribution of the p.Glu386* *IFNAR1* variant on other Pacific islands

We also analyzed an independent cohort of 266 individuals from the geographic transect used to study the peopling history of the Pacific (Bergstrom et al., 2020; Choin et al., 2021), and 1,354 newly sampled individuals from eastern Polynesia (*n* = 764 for the Society Islands; *n* = 211 for the Austral Islands; *n* = 199 for the Marquesas Islands, *n* = 165 for the Tuamotu Islands, and *n* = 15 for the Gambier Islands). We also tested independent population samples from a broader region, including western Polynesia (*n* = 22 from Tonga; *n* = 70 Cook Islands) and Fiji (*n* = 24; Ioannidis et al., 2021). The variant was not observed in most of the Pacific populations studied. This may reflect limitations relating to recruitment criteria, sample size, and the particular archipelagos sampled. However, the variant was present in the Austral Islands (*n* = 211, AF = 0.00474), the Society Islands (*n* = 764, AF = 0.000654), the Marquesas Islands (*n* = 199, AF = 0.00251), the Cook Islands (*n* = 70, AF = 0.0143), and Fiji (*n* = 24, AF = 0.0208; Fig. 4 and Table 2). By contrast, the frequency of G>T in gnomAD 2.1.1 (Karczewski et al., 2020) and TOPMed BRAVO freeze 8 (Taliun et al., 2021) combined is four of a total of 485,756 alleles (AF = 0.00000823). The lack of p.Glu386* detection in several of the Pacific island populations sampled here does not exclude its presence on these islands, because, as indicated by the minor allele frequency (MAF) confidence intervals in Table 2, many of our samples were too small to exclude definitively the possibility of the variant being present. Nevertheless, our findings suggest that the MAF of the allele in these populations is <1%, contrasting with the situation in western Polynesia. Our results therefore suggest that the frequency epicenter of the variant may be western Polynesia (Fig. 4 and Table 2), with this variant being exceedingly rare or absent in the other regions of the world tested. From the data presented here, it is also impossible to draw any firm conclusions about the presence, absence, or frequency of the variant in the unsampled populations, including other populations from eastern Polynesia, New Zealand, Micronesia, and, more generally, other regions of the Pacific.

**Table 2.** Frequency of the *IFNAR1* variant allele and genotypes in Polynesia

				95% CI		Genotype count			
Population	Sample size	Allele count	MAF	Lower	Upper	GG	GT	TT	Expected P(TT)
Europe	34,013	0	0.0000	0.0000	0.0001	34,013	0	0	0.00000
East Asia	2,602	0	0.0000	0.0000	0.0007	2,602	0	0	0.00000
Taiwan	19	0	0.0000	0.0000	0.0918	19	0	0	0.00000
Philippines	40	0	0.0000	0.0000	0.0458	40	0	0	0.00000
New Guinea	17	0	0.0000	0.0000	0.1015	17	0	0	0.00000
Bougainville	11	0	0.0000	0.0000	0.1487	11	0	0	0.00000
Vanuatu	179	0	0.0000	0.0000	0.0106	179	0	0	0.00000
Fiji	24	1	0.0208	0.0011	0.1090	23	1	0	0.00043
Tonga	22	0	0.0000	0.0000	0.0803	22	0	0	0.00000
Samoa	1,285	32	0.0125	0.0088	0.0175	1,253	32	0	0.00016
Cook Islands	70	2	0.0143	0.0039	0.0506	68	2	0	0.00020
Society Islands	764	1	0.0007	0.0000	0.0037	763	1	0	0.00000
Austral Islands	211	2	0.0047	0.0013	0.0171	209	2	0	0.00002
Gambier Islands	15	0	0.0000	0.0000	0.1135	15	0	0	0.00000
Tuamotu Islands	165	0	0.0000	0.0000	0.0115	165	0	0	0.00000
Marquesas Islands	199	1	0.0025	0.0001	0.0141	198	1	0	0.00000

MAF of the g.34725076G>T variant (c.1156G>T) in Polynesia, and number of samples evaluated. 95% binomial confidence intervals are shown. The frequencies of the TT genotype are estimated from the allele frequencies and not from the genotype counts.

## Discussion

We describe here seven affected individuals from five unrelated kindreds of western Polynesian ancestry who experienced severe LAV and viral infections and were found to be homozygous for the same LOF variant of *IFNAR1*. There are now 16 reported patients with AR IFNAR1 deficiency from 12 unrelated kindreds (Abolhassani et al., 2022; Bastard et al., 2021b; Gothe et al., 2022; Hernandez et al., 2019; Khanmohammadi et al., 2022), and almost half (7 of 16) have western Polynesian ancestry. There are also three known patients, from three kindreds, in three different countries with AR IFNAR2 deficiency (Bastard et al., 2021c; Duncan et al., 2015; Passarelli et al., 2020). The range of viral illnesses in these patients with type I IFN receptor deficiency is narrower than predicted from previous studies of type I IFNs, which were widely thought to be essential for host defense against many, if not all, viruses (Duncan et al., 2021; Meyts and Casanova, 2021). The penetrance of severe LAV and WT viral disease appears to be incomplete in patients with IFNAR1 or IFNAR2 deficiency. Severe adverse events following MMR or YFV vaccination have been reported in most cases, but some IFNAR1-deficient individuals may tolerate these vaccines and not be identified due to potential collider or ascertainment bias (Griffith et al., 2020). The severe WT viral diseases occurring in these patients probably include conditions with high penetrance, such as SARS-CoV-2 and critical COVID-19 pneumonia, and some conditions with a lower penetrance, such as HSV-1 and HSE. The mechanisms underlying incomplete penetrance are unknown but may depend on the viral strain, inoculum, and modifier genes governing other antiviral mechanisms, such as genes involved in the type III IFN pathway. IFNAR1 and IFNAR2 deficiencies should be considered in patients with viral illnesses, such as LAV disease, critical COVID-19 pneumonia, and HSE, but also in patients with other, unexplained severe viral illnesses (Casanova and Abel, 2021).

LAV disease may unmask IFNAR1 deficiency, but as clinicians, we nevertheless support the continuation of active MMR immunization for individuals of Polynesian ancestry. In 2019, a measles epidemic in Samoa resulted in 83 deaths, mostly in young unvaccinated children. This outbreak occurred due to low vaccination coverage and ended only after a mass MMR vaccination campaign (Champredon et al., 2020; Craig et al., 2020). No severe adverse events were observed during or after this immunization campaign. This highlights the much higher risk of death in unvaccinated populations due to vaccine-preventable disease than of IFNAR1 deficiency leading to severe complications of MMR vaccination. Individuals with IFNAR1 deficiency have a predicted high susceptibility to the WT measles virus, so a small fraction of severe WT measles cases may actually be due to IFNAR1 deficiency. The impact of IFNAR1 deficiency in this recent epidemic—or in the 1893 Samoan measles epidemic, which resulted in ≥1,000 deaths in a population of 34,500 (Davies, 1894)—is unknown.

The IFNAR1 p.Glu386* LOF variant is exceedingly rare or undetected in studied populations outside the Pacific, but is present at a higher-than-expected frequency in individuals of Polynesian ancestry, including the inhabitants of Samoa and the Cook, Society, Marquesas, and Austral islands. Its observation in Fiji is also given by an individual of Polynesian ancestry (Ioannidis et al., 2021), so despite the complex composition of the island, including populations of mostly Papuan-related ancestry, our findings so far indicate a distribution restricted to Polynesian roots. It is not possible to draw broad conclusions about its frequency in the easternmost parts of eastern Polynesia, New Zealand, Micronesia, and other regions from Near and Remote Oceania based on the data presented here. No pLOF variant of *IFNAR1* or *IFNAR2* has a frequency higher than 10^−4^ in gnomAD, implying that the global frequency of individuals with AR deficiencies of the products of either of these genes is very low, although such genomic databases are known to lack diversity and to be strongly biased toward European ancestries (Sirugo et al., 2019). Like the isolated high prevalence of Huntington disease in Zulia, Venezuela; maple syrup urine disease among the Mennonites of Pennsylvania, USA; or complete achromatopsia on Pingelap, Micronesia, our study suggests that the frequency of IFNAR1 or IFNAR2 deficiency may be much higher in small, geographically circumscribed populations that have experienced strong genetic drift due to serial founder effects, isolation, or bottlenecks. This may explain, for example, the estimated prevalence of *IFNAR1* p.Glu386 homozygosity of 1 in 6,450 individuals of Samoan descent. It is, therefore, now a matter of priority to determine homozygosity rates, penetrance, and clinical presentations in populations of all Polynesian ancestries, including the Aotearoa New Zealand and Cook Island Māori and native Hawaiians, larger samples from eastern Polynesia, the Polynesian diaspora (i.e., Polynesian communities residing outside the Polynesian Triangle), and other communities around the Pacific Rim, including those in Australia, Canada, New Zealand, and the United States. Likewise, studies of the presence of this variant in peoples of Papuan-related ancestry should provide us with information about its broader geographic distribution in the South Pacific region.

Archaeological and cultural records suggest that Samoa was founded ∼2,800 yr ago by cultures capable of long-distance, two-way, open-ocean navigation (Petchey, 2001). We previously reported evidence of an extreme bottleneck in Samoans that lasted for 2,000 yr, ending about 1,000 yr ago, followed by a rapid population expansion (Harris et al., 2020). This suggests that the higher-than-expected allele frequency of *IFNAR1* p.Glu386* among individuals of western or eastern Polynesian ancestries may result from a founder effect followed by an extended bottleneck within which substantial genetic drift may have occurred, coupled with an absence of negative selection against the allele due to the rare or low level of exposure to viruses controlled by type I IFN-dependent immunity in this region before the arrival of Europeans. The frequency of this variant may also have be affected by the population crash accompanying contact with Europeans and the subsequent exponential growth of the population (Harris et al., 2020).

Urgent research and policy priorities will need to be addressed to translate our findings into improvements in health equity for people of Polynesian ancestry, who may be more frequently affected by IFNAR1 deficiency than other populations. Rapid, cost-effective, and accessible diagnostic methods will be required, because IFNAR1 deficiency is difficult to recognize clinically due to the existence of both nonspecific and unknown presentations. In parallel, we will need to harness recent advances in antiviral approaches to provide passive (neutralizing monoclonal antibodies) and active (mRNA vaccination) immunization and treatment (novel antiviral agents). Access to screening, testing, treatment, and follow-up for IFNAR1 deficiency is likely to be inequitable across affected nations and communities, and we call upon well-resourced agencies and organizations to support the establishment of medical and public health infrastructures to enable affected Polynesian communities to benefit equitably from such measures.

Diagnosis in a larger number of kindreds and population studies will facilitate more detailed evaluations of the frequency and clinical phenotypes of IFNAR1-deficient individuals, including the penetrance of each viral illness. These data could then be used to guide decisions about the appropriateness of early screening, and as a basis for prompt diagnosis and potentially life-saving interventions, as has already been clearly demonstrated for neonatal SCID screening (Amatuni et al., 2019; Biggs et al., 2017; Kwan et al., 2014). The early diagnosis of IFNAR1 and other type 1 IFN deficiencies not only would be of direct benefit to the affected individuals, but could also potentially increase confidence in vaccination, thereby resulting in better protection of the community. Approaches to establishing the medical and public health infrastructure necessary to achieve such outcomes must include representatives from affected communities and take into account the inequitable distribution of resources and access. Beyond Polynesians, this study highlights the need to expand population and clinical genetics studies to understudied populations around the globe (Sirugo et al., 2019), particularly isolated populations, in which the frequencies of *IFNAR1* and *IFNAR2* pLOF variants should be explored. Beyond *IFNAR1*, this study highlights the importance of combining clinical and evolutionary genetic studies for delineation of the molecular and cellular basis of human immunity (Quintana-Murci, 2019; Quintana-Murci et al., 2007).

## Materials and methods

### Study and ethics approval

Informed consent was obtained in each country of follow-up, in accordance with local regulations and the requirements for institutional review board (IRB) approval of Rockefeller University and Institut National de la Santé et de la Recherche Médicale (INSERM). Experiments were conducted in the United States and France, in accordance with local regulations and with the approval of the IRB of Rockefeller University and INSERM, respectively. The Soifua Manuia study underwent initial ethical review by the IRB at Brown University and undergoes annual continuing ethical review by the IRB at Yale University. Data analysis activities at the University of Pittsburgh were reviewed by their IRB and were determined to be exempt (IRB #PRO16040077) based on the receipt of only de-identified data. All aspects of the original data collection protocols were reviewed and approved by the Health Research Committee of the Samoan Ministry of Health. All participants provided written informed consent for their participation via forms written in Gagana Sāmoa.

For the collection of French Polynesian samples (eastern Polynesia), all participants provided written informed consent. This work is part of a much broader collaboration (Mata’ea project) between the Institut Louis Malardé (Papeete) and Institut Pasteur (Paris) and received ethics approval from the French Comité de protection de personnes (CPP-OUEST III no. 19.08.60/SICNRIPH 19.07.02.38421) and the Comite d’Ethique de la Polynesie Française (MATAEA ID-CRB2019-AO1793-54/Avis no. 80 CEPF_03/09/2019). Population references for western Polynesia were obtained from samples reported in Ioannidis et al. (2020) with the approval of the Oxford University Tropical Research Ethics Committee (reference no. 537-14) for population genetics and medical studies. Informed consent was obtained from all participants in close coordination with local communities and collaborators, including Nuualofa Tuuau (Samoa), Tamarua Teariki (Cook Islands), and many other liaison officers, whose involvement was essential to ensure a respectful approach to participants and community leaders.

### Cells

Peripheral blood mononuclear cells were isolated by Ficoll-Paque density gradient (GE Life Science) centrifugation. Primary fibroblasts and SV40-immortalized dermal fibroblasts were maintained in DMEM (Thermo Fisher Scientific) supplemented with 10% FBS (Thermo Fisher Scientific). PHA-blasts were cultured in RPMI (Thermo Fisher Scientific) supplemented with 10% FBS (Thermo Fisher Scientific).

### Plasmids

The *IFNAR1* cDNA was inserted into the pGEMT cloning vector (Promega). Site-directed mutagenesis was performed to obtain the indicated mutant constructs. All *IFNAR1* constructs were then subcloned into pCAGGS for overexpression studies. All constructs were resequenced to ensure that no adventitious mutations were generated during the cloning process.

### Western blotting

Fibroblast cells were left untreated or were treated with IFN-α2 (Miltenyi Biotec), IFN-ω (Peprotec), IFN-β (Miltenyi Biotec), or IFN-γ (Imukin, Boehringer Ingelheim) for the times indicated, before lysis. Cells were lysed in NP-40 lysis buffer (280 mM NaCl, 50 mM Tris, pH 8, 0.2 mM EDTA, 2 mM EGTA, 10% glycerol, and 0.5% NP-40) supplemented with 1 mM dithiothreitol, PhosSTOP (Roche), and cOmplete Protease Inhibitor Cocktail (Roche). The protein lysate was subjected to SDS-PAGE, and the resulting bands were transferred to a nitrocellulose membrane, which was probed with unconjugated primary antibodies and secondary antibodies adapted for LI-COR. An anti-GAPDH antibody (Santa Cruz Biotechnology) was used as a loading control. For endogenous IFNAR1, we used an antibody recognizing the IFNAR1 protein at a dilution of 1:1,000 (64G12 custom antibody), whereas, for the protein overexpressed after transfection, we used a polyclonal anti-IFNAR1 antibody recognizing the C-terminus of IFNAR1 (ab45172; Abcam). Antibodies against p-STAT1 (562070; BD Biosciences) and GAPDH (sc-47724; Santa Cruz Biotechnology) were purchased from commercial suppliers. The membrane was incubated overnight at 4°C with the primary antibodies. SuperSignal West Pico Chemiluminescent substrate (Thermo Fisher Scientific) was used to visualize HRP activity, and the resulting signal was detected with an Amersham Imager 600 (GE Life Sciences). The complete unedited blots are shown in the supplemental material.

### Flow cytometry

For measurement of the cell-surface expression of IFNAR1, control or patient EBV-B cells or SV40-fibroblasts were plated in 96-well plates at a density of 5 × 10^5^ cells per well and surface-stained with purified mouse anti-IFNAR1 AA3 mAb (a gift from L. Runkel, Biogen, Inc., Cambridge, MA). Cells stained with AA3 were washed once with PBS and incubated with a biotinylated rat anti-mouse secondary antibody (Thermo Fisher Scientific) for 30 min, before being washed once with PBS and incubated for 30 min with PE-conjugated streptavidin (Thermo Fisher Scientific). The cells were then washed twice with PBS and analyzed by flow cytometry. Data were acquired on a Gallios flow cytometer (Beckman Coulter), and the results were analyzed with FlowJo software (TreeStar).

### RT-qPCR

RNA was isolated from peripheral blood mononuclear cells, fibroblasts, or HEK293T cells with and without plasmid transfection, with a kit, according to the manufacturer’s protocol. We extracted mRNA from the cells with the Cell-to-CT kit (AM1729; Thermo Fisher Scientific), according to the manufacturer’s instructions. RT-qPCR was performed with Applied Biosystems Taqman assays for MX1 and the β-glucuronidase (GUS) housekeeping gene for normalization. Results are expressed according to the ΔΔCt method, as recommended by the kit manufacturer.

### Genotyping

The Soifua Manuia study follows a cohort of adult Samoans who were recruited for a genome-wide association study of adiposity-related phenotypes in Samoa in 2010 (Hawley et al., 2014). Details of DNA extraction for this study were provided by Minster et al. (2016). Of the *n* = 3,475 participants recruited for the study, DNA was available for *n* = 3,119. Of these, *n* = 1,285 participants were selected for whole-genome sequencing via the Trans-Omics in Precision Medicine (TOPMed) Whole-Genome Sequencing Program (Taliun et al., 2021). The allele and genotype counts and frequencies presented here are those from the TOPMed Freeze 9 callset (NHLBI Trans-Omics for Precision Medicine, 2021). There may be a bias in the allele frequencies calculated for research participant samples in which some participants are related. In addition to reporting the counts and frequencies of the entire sequenced sample, we also ascertained a maximum unrelated subset of participants with PRIMUS (Staples et al., 2013). Individuals inferred to be first- or second-degree relatives were labeled as “related” for the ascertainment of this “unrelated” subset. Freeze 8 of the BRAVO variant browser does not include the participants from Soifua Manuia in the allele counts. However, in all TOPMed freezes, the genotypes of Soifua Manuia participants were called together with the genotypes of all other participants. Thus, the observation of 32 alleles in the Soifua Manuia participants and the observation of an absence of these alleles in the remaining TOPMed participants were made simultaneously. For Europeans and East Asians, information was obtained directly from the gnomAD browser. For Polynesians from Tonga, the Cook Islands, and the islands of Fiji, Sanger analysis was performed. For near and western remote Oceanians, we processed the fastq file for Oceanian individuals from Choin et al. (2021) together with the available whole-genome sequences for New Guineans and Bougainville islanders from Bergstrom et al. (2020), as described previously.

### Kindred A, P1

This presentation initiated the investigation of IFNAR1 immunodeficiency in the Pacific. A previously healthy, 12-mo-old girl living in western Polynesia with non-consanguineous Polynesian parents received her first MMR vaccine on day 1. She presented with vaccine site inflammation on day 5 and started oral antibiotics. Further fever and localized swelling led to admission for intravenous antibiotics for presumed abscess. Her vaccine site improved, and there was a transient blanching rash, followed by clinical deterioration with petechial rash, bloody diarrhea, thrombocytopenia, and coagulopathy, requiring fresh frozen plasma and platelet transfusions. All blood cultures were sterile, and dengue serology was negative. On medical history, she had normal growth and previously tolerated live attenuated vaccines BCG and three doses of oral polio vaccine, in addition to scheduled inactivated vaccines. Prior illnesses included conjunctivitis aged 3 mo and a furuncle treated with antibiotics aged 10 mo. On family history, an older brother died at 12 mo of age from overwhelming sepsis 21 d after first MMR as fully described in kindred A, patient 2 (P2) case report.

On day 14, due to continued deterioration, she was transferred to New Zealand and admitted to pediatric intensive care with an oxygen requirement, tachycardia, irritability, hepatosplenomegaly confirmed on abdominal ultrasound, no significant lymphadenopathy, widespread petechiae, and purpura without active bleeding. Her rash was not morbilliform, and the MMR vaccination site was firm and indurated without fluid-filled abscess. Full blood count showed normocytic anemia 81 g/liter, elevated white cell count with left shift, and marked thrombocytopenia with platelets <10 × 10^9^. The peripheral smear showed RBC fragments and thrombocytopenia. Initial coagulation profile, fibrinogen, and ADAMTS-13 were normal. Serum creatinine was normal, and liver transaminases were elevated. Peripheral blood flow cytometry, lymphocyte subsets, and immunoglobulins were also normal. Multiple blood and catheter urine cultures were sterile, and arboviruses were not detected. No LP was performed due to patient instability; however, CT head was normal, and postmortem CSF culture showed no evidence of meningitis. Ferritin was elevated at 4,613 μg/liter, rising to peak ferritin of 38,074 μg/liter. Triglycerides were elevated, fibrinogen was decreased, and complement C3 and C4 were low. Soluble CD25 assay was unavailable.

A diagnosis of HLH was made, and dexamethasone was started on day 14. Broad-spectrum antibiotics and blood products were continued. On day 15, she was intubated and developed progressive circulatory failure requiring inotropes, renal failure requiring continuous filtration, and hepatic failure. She was treated with etoposide with no response, and then alemtuzumab. She developed abnormal neurological signs and died despite maximal supportive care on day 18.

She was PCR positive for MMR in whole blood and nasopharyngeal swab with low cycle time, indicating high viral load. Measles and mumps were both confirmed as vaccine strain (Measles Leningrad-16). Day 14 after MMR, measles IgM was positive, and IgG was negative. HHV6 DNA was PCR positive in whole blood, but HHV6 serology was unavailable. PCR was negative for adenovirus and CMV in plasma and negative for EBV, HSV, and varicella in whole blood. Nasopharyngeal swab and postmortem lung swabs were negative for respiratory viruses.

Postmortem histology showed histiocytic infiltrates of multiple organs consistent with HLH. Multinucleated giant cells, WFCs pathognomic of measles infection were found in multiple organs including lungs, LNs, spleen, liver, thymus, bowel, and adrenals (Fig. 1 B, callosum, and to a lesser extent the cerebellar hemispheres; it was felt to be impossible to determine). Postmortem examination showed no evidence of malignancy. A commercial pan-hematology and pan-immunology next-generation sequencing gene panel identified heterozygous missense variants in XIAP c.337G>G, p.(Gly113Arg), and CXCR4 c.786C>A, p.(Asp262Glu). IFNAR1 was not included in the reported genes.

### Kindred A, P2

P2 was the only sibling of P1. P2 was a previously healthy 12-mo-old male living in Polynesia who died in his domicile country with a diagnosis of overwhelming sepsis 2 yr before P1. He received his first MMR vaccine on day 1 and presented on day 2 with a febrile seizure and respiratory illness and was admitted on broad spectrum antibiotics. He developed fever and a distended abdomen with hepatosplenomegaly, transaminitis, and petechiae. There was anemia, thrombocytopenia, and leukocytosis, and he developed RBC fragments on peripheral smear. Ferritin, triglycerides, and soluble CD25 were unavailable. LP was not performed. Blood cultures were negative.

An exploratory laparotomy on day 16 showed peritoneal free fluid, dilated bowel, multiple enlarged abdominal LNs, and an inflamed appendix and he underwent appendectomy. He continued to deteriorate with high fevers and multiorgan failure, including acute kidney injury, respiratory compromise, shock, and coagulopathy. There was intensive management including invasive ventilation, inotropes, hydrocortisone, blood products, and broad-spectrum antibiotics. He died 21 d after MMR administration.

On medical history, he had normal growth and tolerated live attenuated vaccines: BCG and three doses of oral polio vaccine. There were no prior significant illnesses.

Histology of the appendix was limited by sample degradation but showed giant cells with no definite evidence of measles or HLH. Genomic DNA was isolated from deparaffinized appendix tissue block. Using forward (5′-ATT​CCC​TGA​TTT​CTT​GAG​G-3′) and reverse (5′-AGT​CAG​TGG​TTT​CAA​ATT​AGG-3′) PCR primers, we performed Sanger sequencing and confirmed that the patient was homozygous for IFNAR1 c.1156G>T. Measles was PCR positive on the appendix.

### Kindred B, P3

P3, from kindred B, was a 15-mo-old girl born to non-consanguineous parents of predominantly Tongan and Niuean ancestries. Her great-great-grandfather on the maternal side was of Māori ancestry. History was significant for the development of acute seizures in association with suspected enteroviral meningoencephalitis at 8 wk of age (based on clinical features and the detection of enterovirus in the stool; CSF not obtained). She recovered from this illness with good control of seizures on levitiracetam and was making steady neurodevelopmental progress before her latest hospital presentation.

P3 received the MMR/V vaccination at 15 mo of age as per the New Zealand Immunization Schedule. On day 11 after vaccination, she presented to her local hospital with fever and rash and was found to be encephalopathic. Empirical broad-spectrum antibiotics were commenced. Extensive search for infectious organisms was initially negative. This included viral PCR for CMV, EBV, and adenovirus on serially collected plasma samples, and blood cultures and PCR panels for respiratory viral and atypical pathogens (fungi, *Nocardia*, *Legionella*, *Mycoplasma*, *Chlamydia*, *Pneumocystis jeroveci*, pertussis, CMV, adenovirus, human parainfluenza virus [HPIV]1/2/3, influenza A virus [IAV], influenza B virus [IBV], SARS-CoV2, RSV, and human metapneumovirus [HMPV]). Attempts at obtaining cerebral spinal fluid (CSF) via lumbar puncture were unsuccessful.

By day 17, she had developed features of hyperinflammation or HLH-like illness with persistent fever, cytopenia, hyperferritinemia (peak level 5,224 μg/liter), and elevated sCD25 (peak level 12,940 pg/ml) and had evidence of hemophagocytosis on bone marrow aspirate. She developed aseptic arthritis involving her knee and ankle joints bilaterally. There was no radiologic evidence of central nervous system (CNS) involvement of HLH. However, CNS neuroimaging did demonstrate an incidental finding of a long segment of cervical and thoracic cystic lesions and a chronic communicating hydrocephalus, possibly explaining difficulties with obtaining CSF samples via lumbar puncture.

For the hyperinflammation, P3 received intravenous methylprednisolone for 3 d followed by a weaning course of steroids for a total duration of 2 wk during which time she defervesced, her cytopenias resolved, and sCD25 reduced to 6,758 pg/ml. Her clinical course was complicated by the development of cutaneous varicella reactivation (skin lesion fluid PCR positive for VZV) on day 6 of steroids. This infection responded appropriately to 10 d of acyclovir. She remained persistently encephalopathic and eventually received an extraventricular device (EVD) from which CSF samples could be obtained on day 58. CSF samples were culture negative and PCR negative for HSV1/2, VZV, enterovirus, parechovirus, *Neisseria meningitides*, cryptococcus antigen, CMV, EBV, and *Toxoplasma*. CSF sample was PCR positive for HHV6, but this finding was of uncertain clinical significance.

Around this time, P3 was found to be homozygous for the IFNAR1 p.Glu386* allele on a commercial comprehensive diagnostic primary immune deficiency and cytopenias gene panel. We hypothesized that the presence of an IFNAR1 immune deficiency would explain the patient’s susceptibility to LAV, predisposing to the persistence of a viral encephalitis and retrospectively requested MMR viral PCRs on plasma and CSF samples collected at D54 and D58 respectively. She was found to be PCR positive for vaccine strain measles virus in plasma and vaccine strain measles and mumps viruses in the CSF. Knee joint fluid obtained when she developed arthritis was found to be PCR positive for mumps virus on retrospective testing. She was transferred to a tertiary pediatric hospital pediatric intensive care unit for escalation of care. Further neurological workup revealed stable CNS neuroimaging findings and electroencephalogram (EEG) evidence of encephalopathy with no seizure activity. On D62, she developed unexplained acute respiratory deterioration necessitating intubation and commencement of mechanical ventilation. She remained persistently encephalopathic and unresponsive, and on day 72, she died after medically initiated withdrawal of ventilatory support. A postmortem examination was declined by the family.

P3’s IFNAR1 p.Glu386* variant was confirmed by Sanger. Parental segregation studies demonstrated that the variant was biparentally inherited. Functional studies using donated skin fibroblasts obtained from P3 supported the variant’s loss-of-function nature in homozygous state. Her fully siblings aged 5 and 2 yr are heterozygous carriers of IFNAR1 p.Glu386*. All known carriers in kindred B are healthy and well and tolerated routine childhood immunizations including MMR vaccinations.

Based on the clinical, laboratory, and population genetics information reported in this manuscript, the IFNAR1 c.1156G>T, p.Glu386* variant has been classified as likely pathogenic (ACMG class 4: PVS1_Strong, PS3_Supp, PM3).

### Kindred C, P4 and P5

P4, from kindred C, was a 13-mo-old boy born at 24 wk gestation to non-consanguineous parents of Niuean ancestry. His premorbid conditions included premature birth associated chronic lung disease, which was stable on low-flow home oxygen (0.25 liter/min), and CNS changes including a left-sided porencephalic cyst and periventricular white matter changes without seizures. He was followed by neurodevelopmental specialists and was making satisfactory neurodevelopmental progress. In the postnatal period, he spontaneously recovered from a CMV infection. He tolerated routine childhood immunizations including rotavirus vaccines in his first year of life.

On day 10 after receiving his first dose of MMR immunization, he developed a vaccine site reaction and was commenced on a course of antibiotics for presumed cellulitis. He was coincidentally found to have a lower respiratory tract infection with respiratory tract secretion PCR positive for HPIV-1. On day 14, he was admitted to the pediatric intensive care unit for management of acute neurological and respiratory deterioration including status epilepticus. He was encephalopathic, with CSF pleocytosis and EEG changes consistent with encephalopathy. Neuroimaging revealed no acute changes from previous studies. He developed features of hyperinflammation including persistent fevers, bicytopenia (nadir hemoglobulin level 82 g/liter, nadir platelet count 32 × 10^6^/liter), hepatomegaly, biochemical hepatitis (peak ALT 202 U/liter), hyperferritinemia (1,861 μg/liter), and elevated sCD25 (15,536 pg/ml). Extensive search for infectious organisms in the peripheral blood (blood cultures, PCR for EBV, adenovirus, HHV6, CMV), respiratory tract samples (PCR for adenovirus, CoV, MERS-CoV, SARS-CoV2, HMPV, rhinovirus (RV)/enterovirus (EV), IAV, IBV, HPIV1–4, RSV, *Bordetella*, *Chlamydophila*, *Mycoplasma*), and CSF (stain and culture; PCR for HHV6, *Escherichia coli*, HiB, *Listeria*, *Neisseria*, *Streptococcus*, CMV, EV, HSV1, HSV2, human parechovirus, VZV, *Cryptococcus*) were all negative. Given the temporal association between exposure to LAVs and onset of symptoms, we hypothesized that his aggressive clinical course was mediated by the presence of LAVs. CSF sample collected on day 19 returned PCR positive for vaccine strain mumps virus, and a peripheral blood sample collected on day 22 was positive for vaccine-strain measles virus (persistently detectable in peripheral circulation to at least day 45) and mumps virus (persistently detectable in peripheral circulation to at least day 56). We commenced intravenous immunoglobulin (1 g/kg), vitamin A and intravenous ribavirin. Following this, there was gradual improvement of his fever, cytopenias, and hepatitis. However, there was minimal change in his respiratory and neurological status. In week 6, P4 developed biochemical hepatitis attributed to CMV reactivation. There was no acute respiratory or neurological deterioration. He was treated with (val)ganciclovir for 3 wk, during which time the hepatitis resolved. P4 had a protracted recovery from the acute encephalitis and acute chronic respiratory distress syndrome. From week 7 onward, he was more alert and responsive and had a normal EEG on day 56. Noninvasive respiratory support with Hi-Flo was weaned successfully from week 10 onward toward baseline requirements. In week 16 he was stable enough to have short periods of leave from the hospital.

The following week, P4 developed acute bronchiolitis. Respiratory tract sample was PCR positive for RSV (negative for other pathogens including PIV1/2/3, HMPV, adenovirus, RV, IAV, IBV, SARS-CoV2, *Legionella* species, *Mycoplasma pneumoniae*, *Chlamydophila pneumoniae*, *P. jiroveci*, *Bordetella pertussis*). Peripheral blood was PCR negative for MMR viruses. Despite aggressive intensive care support and oscillatory ventilation support, P4 died of acute respiratory distress syndrome 4 d after readmission. A postmortem examination was declined by the family.

Patient 5 (P5) is P4’s full biological brother and is alive at 7 yr of age. P5 has tetralogy of Fallot, which was surgically corrected at age 6 mo. In early infancy, he had recurrent respiratory tract infections and was briefly hospitalized four times for bronchiolitis. At 15 mo of age, he was hospitalized for viral meningoencephalitis and hyperinflammation, diagnosed at the time as “atypical Kawasaki disease,” 17 d after he received his first dose of MMR immunization. He had fevers, coryza, cough, irritability, maculopapular rash, nonpurulent conjunctivitis, and mild peripheral edema. He had preserved peripheral blood cell lines but had a biochemical hepatitis (peak ALT 134 U/liter, peak AST 127 U/liter). CSF sample showed 54 white blood cells (90% lymphocyte predominance) and seven RBCs. CSF cultures and a standard viral PCR panel (HSV, VZV, enterovirus, parechovirus, EBV, *Streptococcus pneumoniae*, *Neisseria meningitidis*) were negative. He was hospitalized for 6 d and received intravenous antimicrobial therapy (cefotaxime and acyclovir), immunomodulatory dose of intravenous immunoglobulin (1 g/kg), and aspirin, which was continued for 6 wk. At 2 yr of age, he was hospitalized for 10 d with meningitis accompanied by PCR detection of enterovirus in the CSF. CSF was culture negative and PCR negative for HSV, VZV, parechovirus, EBV, *S. pneumoniae*, *N. meningitides*. He was later confirmed to have a unilateral high-frequency sensorineural hearing loss. He subsequently tolerated his second dose of MMR given at 4 yr of age and has not had any further significant infections. He has not received varicella vaccination. He attends a mainstream school and receives speech language therapy and resource teacher support for a mild learning difficulty.

We evaluated for and confirmed homozygosity for IFNAR1 p.Glu386* allele in P4 and P5 by Sanger sequencing. Parental segregation studies demonstrated that P4’s and P5’s IFNAR1 p.Glu386* variant are biparentally inherited. A commercial comprehensive primary immune deficiency diagnostic gene panel excluded the presence of other pathogenic variants in P4.

### Kindred D, P6

P6 from Kindred D is a 4-yr-old boy born in Australia to Niuean and Tongan parents, who has four older siblings, all of whom are well, with two having a history of language disorders. He was born at term with no neonatal concerns and normal newborn hearing screen (otoacoustic emissions). He suffered recurrent viral pneumonitis over the first year of life but was otherwise felt to be developing normally. At 14 mo, some 5 d after receiving the MMR vaccine, he developed fever, lymphadenopathy, and a generalized blanching rash and progressed to have arching movements and lethargy with increased irritability but no localizing signs. 2.5 wk into this illness, he was found to have a CSF pleocytosis (polymorphs = 23 × 10^6^/liter; mononuclear = 43 × 10^6^/liter) consistent with viral encephalitis. At 4 wk after MMR he was positive for measles virus on NPA, while brain MRI performed during the acute phase was said to be normal. He did not receive amino glycoside antibiotics. The encephalitis self-remitted after 4 wk, but following the hospital admission, his family felt that his behavior had changed, with concerns about developmental delay, including walking later than his siblings at 18 mo, and no clear words by 21 mo of age when he was due to see Speech Pathology. However, at 21 mo he developed viral pneumonitis, requiring intensive care and venovenous ECMO, with RSV type A, parainfluenza type 3, rhinovirus, and bocavirus on NPA. Following this admission, his family felt that he returned to his pre–intensive care level of function, and shortly thereafter he was diagnosed with profound bilateral sensorineural hearing loss with no response in either ear on auditory brainstem-evoked responses. MRI scan demonstrated a dysplastic right vestibule and absent right lateral semicircular canal. He was also diagnosed with global developmental delay and autistic spectrum disorder, a finding that has been reinforced by more recent neurodevelopmental assessment performed at age 4 yr and 8 mo following the placement of cochlear implants. He demonstrated skills less than the first centile in all modalities assessed, with a functional level <18 mo based on clinical review of items completed on a standardized developmental assessment tool. The patient’s engagement was limited, and this will be reviewed via a normed functional parent questionnaire which is pending, but parental history supports these findings. Behavioral review demonstrated a significant deficit in social communication skills and restricted and repetitive behaviors consistent with DSM-5 criteria for a diagnosis of autism spectrum disorder. He currently needs substantial support with his social communication (level 2) and very substantial support for his behaviors (level 3). This will be reviewed following intervention, which to date has been limited. More recent brain MRI has demonstrated multiple foci of “blooming,” in keeping with chronic micro-bleeds, predominantly involving the bilateral cerebral subcortical white matter, splenium of the corpus callosum, and to a lesser extent, the cerebellar hemispheres. It was felt to be impossible to determine whether these were related to sequelae of the encephalitis or the pneumonitis requiring ECMO. He has not had further significant infections in the last 21 mo, and his basic adaptive immune screen is normal including immunoglobulin levels. He is above the 95th centile for height and weight. SNP array at age 4 yr showed that despite no history of consanguinity, 8% of his DNA represented long continuous segments of homozygosity consistent with identity by descent, including across the chr22q21.3–22q22.2 region which includes the IFNAR1 gene locus. He was then diagnosed with homozygous IFNAR1 deficiency by targeted Sanger sequencing. He is awaiting WES to exclude an additional/alternative etiology to his developmental disorders and hearing loss.

### Griffiths III – Griffiths Scale of Child Developmental 3rd edition scores

Griffiths Scale scores are provided in Table S5.

### Kindred D, P7

P7 from Kindred E is a 13-yr-old girl born in New Zealand to non-consanguineous parents of Samoan ancestry following an uneventful pregnancy at term who has six older siblings who are well. She had hospital presentations for a urinary tract infection and two episodes of bronchiolitis in the first 6 mo of life. She presented at 10 mo of age with Hib bacteremia and meningitis (she had not been vaccinated prior). This was complicated by seizures and bilateral infected subdural hygromas requiring multiple washouts. At 12 mo old, 2 wk after discharge from the Hib illness–associated admission she represented with rapidly progressive respiratory and multiorgan failure requiring 9 d of ECMO support. RSV was identified on PCR and immunofluorescence. Bronchioalveolar lavage also isolated *Candida* (one colony), and CMV PCR was strongly positive (7.0 log copies/ml, negative in blood). She was initially treated with high-dose trimethoprim with sulfamethoxazole, caspofungin, palivizumab, and intravenous immunoglobulin.

She relocated to Australia at age 3 and was lost to medical follow-up there. She received vaccination with MMR + MMRV at 4 yr of age without complication. She remained relatively well until she presented at age 7 yr with acute respiratory distress syndrome and multiorgan failure requiring inotropic support. She was intubated for a total of 33 d requiring escalation to high-frequency oscillation ventilation and nitric oxide. Imaging showed extensive air space opacification and background extensive bronchiectasis. No causative organism was identified.

At 8 yr of age, she was admitted with an exacerbation of bronchiectasis with adenovirus and human metapneumovirus identified on NPA. She has had a subsequent admission for pulmonary optimization and remains on prophylactic azithromycin but has not had further acute infective episodes.

In addition to the severe life-threatening infection history, she has a deletion of ∼1.7 Mb on the long arm of one chromosome 17 at cytogenic band q12 and one long continuous stretch of homozygosity (7.2 Mb) on the X chromosome. She is known to have a right renal dysplasia with a pelvic-ureteric junction obstruction. Common features of people with deletions of 17q12 include renal abnormalities and neurocognitive and neurobehavioral problems (Loirat et al., 2010; Moreno-De-Luca et al., 2010; Nagamani et al., 2010). She has global developmental delay. It is unclear whether this is due to her chromosomal deletion or her history of severe infections and seizures in early life. She is allergic to crustaceans and has some eczema. Previous testing on 112-gene WES panel reported a heterozygous variant of uncertain significance in CD3D c.418C>A (p.Gln140Lys). The finding of homozygosity for IFNAR1 p.Glu386* was made on testing 421-gene WES panel. No other variants were reported.

### Online supplemental material

Fig. S1 shows the genetic study of the patients. Fig. S2 shows the study of XIAP in the patients from kindred A. Fig. S3 shows the study of mutant IFNAR1. Fig. S4 shows the population genetics of IFNAR1 based on public databases. Table S1 shows the immunological evaluation of the patients. Table S2 shows the infectious diseases evaluation of patients after exposure to LAVs. Table S3 shows the summary of treatments given during admission for LAV infection. Table S4 shows the genetic evaluation of the patients. Table S5 shows Griffiths Scale of Child Developmental 3rd edition scores.

## Supplementary Material

Table S1 shows the immunological evaluation of the patients.

Table S2 shows the infectious diseases evaluation of patients after exposure to LAVs.

Table S3 shows the summary of treatments given during admission for LAV infection.

Table S4 shows the genetic evaluation.

Table S5 shows Griffiths Scale of Child Developmental 3rd edition scores.

SourceData F2 contains original blots for Fig. 2.
