# Supplementary material for: A loss-of-function IFNAR1 allele in Polynesia underlies severe viral diseases in homozygotes
Source: J Exp Med. 2022 Apr 20;219(6):e20220028. doi: 10.1084/jem.20220028 (PMC9026234; doi:10.1084/jem.20220028)
Supplement: Table S1 — shows the immunological evaluation of the patients. [file JEM_20220028_TableS1.docx]

| **Table S1. Immunological evaluation of the patients** |  |  |  |  |  |  |  |
| --- | --- | --- | --- | --- | --- | --- | --- |
| **Country** | **NZ** | **NZ** | **NZ** | **NZ** | **NZ** | **Australia** | **Australia** |
| **Year diagnosed** | **2018** | **2018** | **2020** | **2021** | **2021** | **2021** | **2021** |
| **Kindred** | **A** | **A** | **B** | **C** | **C** | **D** | **E** |
| **Patient** | **1** | **2** | **3** | **4** | **5** | **6** | **7** |
| Age at presentation after exposure to MMR/V (months of age) | 12 | 12 | 15 | 13 | 16 | 14 | N/A |
| **Lymphocyte subsets (absolute counts)** | | | | | | | |
| CD3+ (×10^6^/liter) | 1,625 | ND | 1,257 | 3,015 | ND | NR | 2290 |
| CD4+ (×10^6^/liter) | 1,032 | ND | 772 | 2,056 | ND | NR | 1100 |
| CD8+ (×10^6^/liter) | 575 | ND | 474 | 916 | ND | NR | 1000 |
| CD19+ (×10^6^/liter) | 1,238 | ND | 206 | 188 | ND | NR | 500 |
| CD16+/56+ (×10^6^/liter) | 267 | ND | 75 | 50 | ND | NR | 310 |
| Naïve CD45RA+/CD62L+/CD4+ (% of CD4+) | ND | ND | 85.7 | 83.8 | ND | NR | ND |
| Naïve CD45RA+/CD62L+/CD8+ (% of CD8+) | ND | ND | 81.1 | 77.7 | ND | NR | ND |
| **B cell function** | | | | | | | |
| IgG (g/liter) | 9.8 | ND | 15.2 | 11.5 | ND | 7.13 | 19.1 |
| IgA (g/liter) | 1.6 | ND | 0.24 | 0.44 | ND | 1.08 | 4.3 |
| IgM (g/liter) | 1.2 | ND | 3.2 | 4.4 | ND | 1.22 | 1.9 |
| Measles IgM | +ve D+15 | ND | ND | ND | ND | NR | NR |
| Measles IgG | -ve D+15 | ND | +ve D+62 | -ve D+20 | ND | NR | NR |
| Mumps IgG | ND | ND | +ve D+62 | ND | ND | NR | NR |

NA, not applicable; ND, not done; NR, not recorded, NZ, New Zealand.
