# Supplementary material for: A loss-of-function IFNAR1 allele in Polynesia underlies severe viral diseases in homozygotes
Source: J Exp Med. 2022 Apr 20;219(6):e20220028. doi: 10.1084/jem.20220028 (PMC9026234; doi:10.1084/jem.20220028)
Supplement: Table S2 — shows the infectious diseases evaluation of patients after exposure to LAVs. [file JEM_20220028_TableS2.docx]

| **Table S2. Infectious diseases evaluation of patients after exposure to live attenuated viruses** | | | | | | | |
| --- | --- | --- | --- | --- | --- | --- | --- |
| **Patient** | **1** | **2** | **3** | **4** | **5** | **6** | **7** |
| **Viruses** | | | | | | | |
| **Peripheral blood** | | | | | | | |
| PCR +ve | MeV^, MuV^, RuV, HHV-6 (D+15) | ND | MeV^ (D+54), EBV 397 IU/ml (D+54) | MeV^ (D+22), MuV^ (D+22), CMV (D+25) | ND | ND | NA |
| PCR -ve | CMV, EBV, ADV, HSV-1, HSV-2, VZV | ND | MuV, RuV, CMV, ADV, HHV-6 | EBV, ADV, HHV-6, HSV, VZV | ND | ND | NA |
| **CSF** | | | | | | | |
| PCR +ve | ND | ND | MeV^, MuV^, HHV-6 (D+58) | MuV^ (D+19) | (none) | ND | NA |
| PCR -ve | ND | ND | HSV1/2, VZV, EV, PV, CMV, EBV | HSV1/2, VZV, EV, PV, CMV, HHV-6, MeV, RuV | EV, HSV, VZV, PV, EBV | ND | NA |
| **Upper respiratory tract and/or tracheal** | | | | | | | |
| PCR +ve | MeV, MuV, RuV (D+14) | ND | (none) | HPIV-1 (D+10); MeV (D+18), MuV (D+18), RuV (D+18), CMV (D+25) | CMV (D+18) | MeV (week 4) | NA |
| PCR -ve | HPIV-1/2/3, RSV, HMPV, ADV, RV, EV, IAV, IBV, CMV | ND | HPIV-1/2/3/4, RSV, HMPV, ADV, RV, EV, IAV, IBV, CMV, SARS-CoV2, Bocavirus, PV | HPIV-1/2/3/4, RSV, HMPV, ADV, CoV, MERS-CoV, SARS-CoV2, RV, EV, IAV, IBV | HPIV-1/2/3, RSV, HMPV, ADV, RV, EV, IAV, IBV | ND | NA |
| **Skin swab** | | | | | | | |
| PCR +ve | ND | ND | VZV (D+31) | ND | ND | ND | NA |
| PCR -ve | ND | ND | HSV | ND | ND | ND | NA |
| **Joint fluid** |  |  |  |  |  |  |  |
| PCR +ve | ND | ND | MuV (D+24) | ND | ND | ND | NA |
| PCR -ve | ND | ND | (none) | ND | ND | ND | NA |
| **Bacteria and others** | | | | | | | |
| **Peripheral blood** | | | | | | | |
| Culture | Negative | Negative | *Haemophilus* (non-typeable; D+25), *Gordonia* species (D+34) | *Staphylococcus epidermidis* (D+18) | Negative | ND | NA |
| **Upper respiratory tract and/or tracheal** | | | | | | | |
| Culture | ND | ND | *Stenotrophomonas maltophilia* (D+58) | Negative | ND | ND | NA |
| PCR +ve | ND | ND | PJP (D+58) | (none) | (none) | ND | NA |
| PCR -ve | ND | ND | *Legionella* | *B. pertussis*, *Chamydophila*, *Mycoplasma* | *B. pertussis* | ND | NA |
| **CSF** | | | | | | | |
| Culture | ND | ND | Negative | Negative | Negative | ND | NA |
| PCR +ve | ND | ND | (none) | (none) | (none) | ND | NA |
| PCR -ve | ND | ND | *N. meningitidis*, *Cryptococcus*, Toxoplasma | *S. pneumoniae*, *N. meningitidis*, *Cryptococcus*, *Listeria*, *E. coli*, *Haemophilus influenzae* B | *S. pneumoniae*, *N. meningitidis* | ND | NA |
| **Skin swab** | | | | | | | |
| Culture | ND | ND | Negative | ND | ND | ND | NA |
| **Joint fluid** | | | | | | | |
| Culture | ND | ND | Negative | ND | ND | ND | NA |

Day 1, day of exposure to LAV; MeV, measles virus; MuV, mumps virus; NA, not applicable; ND, not done; RuV, rubella virus; HHV-6, human herpes virus-6; PV, parechovirus.

^Vaccine strain confirmed on genotyping.
