# Supplementary material for: A loss-of-function IFNAR1 allele in Polynesia underlies severe viral diseases in homozygotes
Source: J Exp Med. 2022 Apr 20;219(6):e20220028. doi: 10.1084/jem.20220028 (PMC9026234; doi:10.1084/jem.20220028)
Supplement: Table S3 — shows the summary of treatments given during admission for LAV infection. [file JEM_20220028_TableS3.docx]

| **Table S3. Summary of treatments given during admission for live attenuated viral infection** | | | | |  |  |  |
| --- | --- | --- | --- | --- | --- | --- | --- |
| **Country** | **NZ** | **NZ** | **NZ** | **NZ** | **NZ** | **Australia** | **Australia** |
| **Year diagnosed** | **2018** | **2018** | **2020** | **2021** | **2021** | **2021** | **2021** |
| **Kindred** | **A** | **A** | **B** | **C** | **C** | **D** | **E** |
| **Patient** | **1** | **2** | **3** | **4** | **5** | **6** | **7** |
| **Antiviral agents** | **N** | **N** | **Y** | **Y** | **Y** | NR | NA |
| Ribavarin |  |  | N | Y | N |  |  |
| Aciclovir |  |  | Y | N | Y |  |  |
| Ganciclovir |  |  | N | Y | N |  |  |
| Oseltamivir |  |  | N | N | N |  |  |
| Other antivirals |  |  | N | N | N |  |  |
| **Antiviral antibodies** | **N** | **N** | **Y** | **Y** | **Y** | NR | NA |
| Pooled polyclonal antibodies (IVIg) |  |  | Y | Y | Y |  |  |
| Neutralising monoclonal antibodies (e.g., ZIG) |  |  | N | N | N |  |  |
| **Immune modulation/suppression** | **Y** | **N** | **Y** | **Y** | **Y** | NR | NA |
| IVIg (high dose) | N |  | Y | Y | Y |  |  |
| Dexamethasone/methylprednisolone | Y |  | Y | N | N |  |  |
| Etoposide | Y |  | N | N | N |  |  |
| Alemtuzumab | Y |  | N | N | N |  |  |
| **Others** | **Y** | **Y** | **Y** | **Y** | **Y** | NR | NA |
| Antibiotics | Y | Y | Y | Y | Y |  |  |
| Antifungals | N | N | Y | N | N |  |  |
| **Death** | **Y** | **Y** | **Y** | **Y** | **N** | **N** | **N** |
| Days post-exposure to LAV | 18 | 21 | 72 | 127^ | Alive | Alive | Alive |

Day 1, day of exposure to LAV; IVIg, intravenous immunoglobulin; NA, not applicable; NR, not recorded; NZ, New Zealand; N, no; Y, yes.

^Died of fatal RSV +ve ARDS.
