# Supplementary material for: A loss-of-function IFNAR1 allele in Polynesia underlies severe viral diseases in homozygotes
Source: J Exp Med. 2022 Apr 20;219(6):e20220028. doi: 10.1084/jem.20220028 (PMC9026234; doi:10.1084/jem.20220028)
Supplement: Table S4 — shows the genetic evaluation. [file JEM_20220028_TableS4.docx]

**Table S4. Genetic evaluation**

| **Patient** | **1** | **2** | **3** | **4** | **5** | **6** | **7** |
| --- | --- | --- | --- | --- | --- | --- | --- |
| **Genetic variants identified by commercial NGS** | **BpG Primary Immunodeficiency Panel Plus 274 genes (2018)*** | **ND** | **BpG Comprehensive Immune and Cytopenia Panel Plus 642 genes (2020)** | **Invitae Primary Immunodeficiency Panel 407 genes (2021)** | **ND** | **ND** | **Commercial NGS Labs^,^^,^^^** |
|  | XIAP c.337G>C, p.Gly113Arg (Het, VUS) |  | IFNAR1 c.1156G>T, p.(Glu386*) (Hom, VUS) | IFNAR1 c.1156G>T, p.(Glu386*) (Hom, VUS) |  |  | IFNAR1 c.1156G>T, p.(Glu386*) (Hom, VUS)^ |
|  | CXCR4 c.786C>A, p.Asp262Glu (Het, VUS) |  | DOCK8 c.3234+2T>C (Het, likely pathogenic) | ADAM17 c.2416G>C (p.Ala806Pro) (Het, VUS) |  |  | CD3D 418C>A (p. Gln140Lys) (Het, VUS)^^ |
|  |  |  | DOCK8 c.3134C>T, p.(Ala1045Val) (Het, VUS) | ANKZF1 c.485C>T (p.Pro162Leu) (Het, VUS) |  |  | Deletion in chromosome region 17q12Arr(hg19): 17q12 (34,618,594-36, 358, 102)^^^ |
|  |  |  | CARD11 c.3095A>G, p.(Asn1032Ser) (Het, VUS) | CARD14 c.2517G>C (p.Lys839Asn) (Het, VUS) |  |  | Long stretch of homozygosity (7.2Mb) in X chromosome^^^ |
|  |  |  | UNC13D c.610A>G, p.(Met204Val) (Het, VUS) | CARMIL2 c.1370T>C (p.Phe457Ser) (Het, VUS) |  |  |  |
|  |  |  | EPG5 c.5929G>C, p.(Glu1977Gln) (Het, VUS) | FPR1 c.301G>A (p.Val101Ile) (Het, VUS) |  |  |  |
|  |  |  | LIG1 c.1645C>T, p.(Arg549Trp) (Het, VUS) | GUCY2C c.3035C>T (p.Thr1012Ile) (Het, VUS) |  |  |  |
|  |  |  | WDR1 c.685G>A, p.(Gly229Arg) (Het, VUS) | HPS4 c.817C>A (p.Pro273Thr) (Het, VUS) |  |  |  |
|  |  |  |  | ITGAM c.890G>A (p.Arg297His) (Het, VUS) |  |  |  |
|  |  |  |  | LYST c.10526G>A (p.Arg3509Gln) (Het, VUS) |  |  |  |
|  |  |  |  | NLRP1 c.2743G>A (p.Ala915Thr) (Het, VUS) |  |  |  |
|  |  |  |  | VPS13B c.9500T>C (p.Met3167Thr) (Het, VUS) |  |  |  |
| ***IFNAR1*-targeted Sanger sequencing** | **Paris** | **LabPlus, Auckland** | **Canterbury Health Laboratories, Christchurch** | **LabPlus, Auckland** | **LabPlus, Auckland** | **LabPlus, Auckland** | **NR** |
| Patient | **IFNAR1 c.1156G>T, p.(Glu386*) (Hom)** | **IFNAR1 c.1156G>T, p.(Glu386*) (Hom)** | **IFNAR1 c.1156G>T, p.(Glu386*) (Hom)** | **IFNAR1 c.1156G>T, p.(Glu386*) (Hom)** | **IFNAR1 c.1156G>T, p.(Glu386*) (Hom)** | **IFNAR1 c.1156G>T, p.(Glu386*) (Hom)** | **IFNAR1 c.1156G>T, p.(Glu386*) (Hom)** |
| Father | IFNAR1 c.1156G>T, p.(Glu386*) (Het) |  | IFNAR1 c.1156G>T, p.(Glu386*) (Het) | IFNAR1 c.1156G>T, p.(Glu386*) (Het) | IFNAR1 c.1156G>T, p.(Glu386*) (Het) |  |  |
| Mother | IFNAR1 c.1156G>T, p.(Glu386*) (Het) |  | IFNAR1 c.1156G>T, p.(Glu386*) (Het) | IFNAR1 c.1156G>T, p.(Glu386*) (Het) | IFNAR1 c.1156G>T, p.(Glu386*) (Het) |  |  |
| Unaffected full sibling 1 |  |  | IFNAR1 c.1156G>T, p.(Glu386*) (Het) |  |  |  |  |
| Unaffected full sibling 2 |  |  | IFNAR1 c.1156G>T, p.(Glu386*) (Het) |  |  |  |  |

ND, not done; NR, not recorded.

*BpG Primary Immunodeficiency Panel Plus 274 genes (2018) - did not include *IFNAR1*.

^Pathology Queensland Whole Exome Primary immunodeficiency panel 421 genes (2021).

^^Fulgent Primary immunodeficiency panel 112 genes panel (2017).

^^^Victorian Clinical Genetics Service Cytogenetics (2015).
