# Supplementary material for: A loss-of-function IFNAR1 allele in Polynesia underlies severe viral diseases in homozygotes
Source: J Exp Med. 2022 Apr 20;219(6):e20220028. doi: 10.1084/jem.20220028 (PMC9026234; doi:10.1084/jem.20220028)
Supplement: Table S5 — shows Griffiths Scale of Child Developmental 3rd edition scores. [file JEM_20220028_TableS5.docx]

**Griffiths III – Griffiths Scale of Child Developmental 3^rd^ Edition Scores**

| Scale | Developmental Age (months) | Raw score | Scaled Score | Developmental Quotient (DQ) & 95% CI | %ile | Stanine |
| --- | --- | --- | --- | --- | --- | --- |
| A. Foundations of Learning | 27 | <32 | 0 | <50 | 50 | 1 |
| B. Language and Communication | 5 | <33 | 0 | <50 | 50 | 1 |
| C. Eye and Hand Coordination | 23 | <31 | 0 | <50 | 50 | 1 |
| D. Personal-Social-Emotional | 17 | <33 | 0 | <50 | 50 | 1 |
| E. Gross Motor | Not administered |  |  |  |  |  |
| General Development (GD) | Not calculated |  |  |  |  |  |
