# Supplementary figures and images for: A loss-of-function IFNAR1 allele in Polynesia underlies severe viral diseases in homozygotes
Source: J Exp Med. 2022 Apr 20;219(6):e20220028. doi: 10.1084/jem.20220028 (PMC9026234; doi:10.1084/jem.20220028)

Figure 2 – B

Uncropped gel

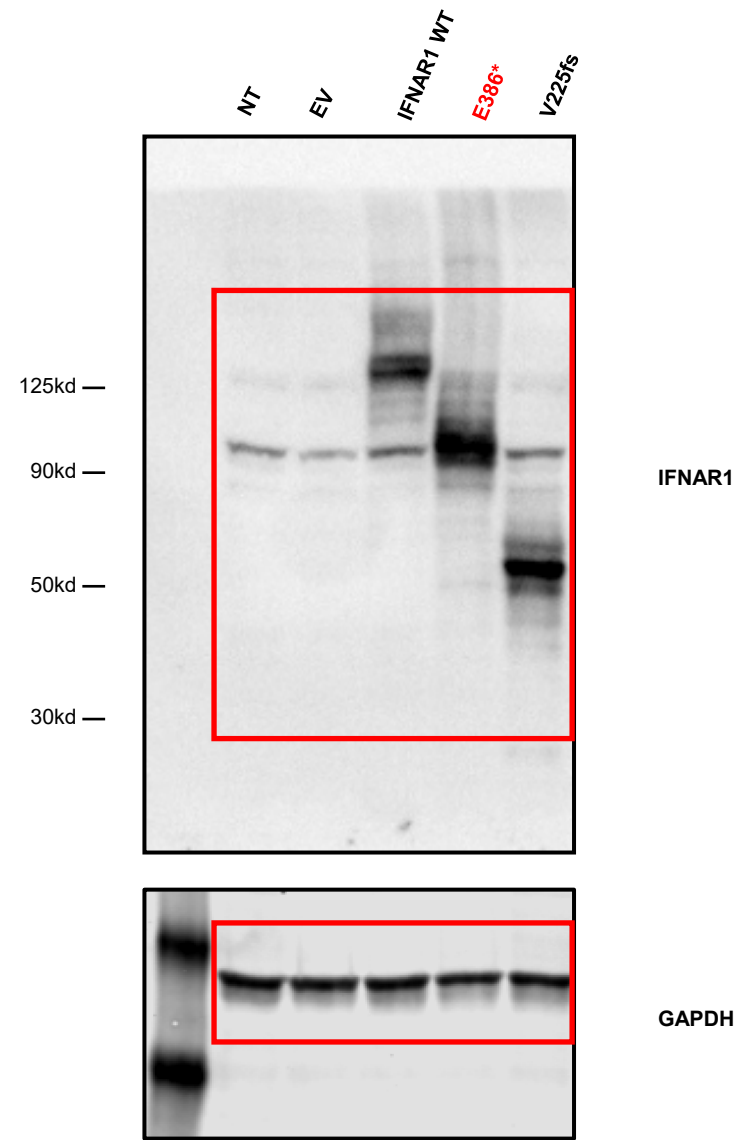

Cropping zone

Supplement: SourceData F2 — contains original blots for Fig. 2. [file JEM_20220028_SourceDataF2.pdf]
